# A *Toxoplasma* MORN1 Null Mutant Undergoes Repeated Divisions but Is Defective in Basal Assembly, Apicoplast Division and Cytokinesis

**DOI:** 10.1371/journal.pone.0012302

**Published:** 2010-08-19

**Authors:** Alexander Lorestani, Lilach Sheiner, Kevin Yang, Seth D. Robertson, Nivedita Sahoo, Carrie F. Brooks, David J. P. Ferguson, Boris Striepen, Marc-Jan Gubbels

**Affiliations:** 1 Department of Biology, Boston College, Chestnut Hill, Massachusetts, United States of America; 2 Department of Cellular Biology and Center for Tropical and Emerging Global Diseases, University of Georgia, Athens, Georgia, United States of America; 3 Nuffield Department of Clinical Laboratory Science, University of Oxford, John Radcliffe Hospital, Oxford, United Kingdom; University of Minnesota, United States of America

## Abstract

The membrane occupation and recognition nexus protein 1 (MORN1) is highly conserved among apicomplexan parasites and is associated with several structures that have a role in cell division. Here we dissected the role of MORN1 using the relatively simple budding process of *Toxoplasma gondii* as a model. Ablation of MORN1 in a conditional null mutant resulted in pronounced defects suggesting a central role for MORN1 in apicoplast segregation and in daughter cell budding. Lack of MORN1 resulted in double-headed parasites. These Janus-headed parasites form two complete apical complexes but fail to assemble a basal complex. Moreover, these parasites were capable of undergoing several more budding rounds resulting in the formation of up to 16-headed parasites conjoined at the basal end. Despite this segregation defect, the mother's cytoskeleton was completely disassembled in every budding round. Overall this argues that successful completion of the budding is not required for cell cycle progression. None of the known basal complex components, including a set of recently identified inner membrane complex (IMC) proteins, localized correctly in these multi-headed parasites. These data suggest that MORN1 is essential for assembly of the basal complex, and that lack of the basal complex abolishes the contractile capacity assigned to the basal complex late in daughter formation. Consistent with this hypothesis we observe that MORN1 mutants fail to efficiently constrict and divide the apicoplast. We used the null background provided by the mutant to dissect the function of subdomains of the MORN1 protein. This demonstrated that deletion of a single MORN domain already prevented the function of MORN1 whereas a critical role for the short linker between MORN domains 6 and 7 was identified. In conclusion, MORN1 is required for basal complex assembly and loss of MORN1 results in defects in apicoplast division and daughter segregation.

## Introduction

The obligate intracellular parasite *Toxoplasma gondii* is a member of the phylum Apicomplexa. Several apicomplexans cause severe disease in humans (malaria, toxoplasmosis, cryptosporidiosis) while others cause economic losses to livestock (coccidiosis, babesiosis, theileriosis). *T. gondii* in particular affects immunosuppressed individuals giving rise to life-threatening encephalitis in AIDS patients. In pregnant women toxoplasmosis can lead to congenital infection causing a variety of birth defects. Clinical disease is associated with the tachyzoite life stage causing tissue lesions by rapid proliferation [1]. *Toxoplasma* divides through a very distinct internal division mechanism termed endodyogeny, wherein two parasites are assembled within a mother parasite [2], [3]. Daughter assembly is driven by the assembly of the cortical cytoskeleton, which consists of three major components; sub-pellicular microtubules, a meshwork of intermediate-filament like proteins, and a series of flattened membrane sacs known as alveoli; the latter two make up the inner membrane complex or IMC [4], [5], [6], [7], [8], [9], [10]. In the final stages of cell division the mother's cytoskeleton disassembles in a tightly orchestrated apical to basal direction. In this process, the mother's plasma membrane is transferred to the daughters, which emerge with their apical ends first [3], [9], [11].

The membrane occupation and recognition nexus protein 1 (MORN1) is highly conserved among apicomplexan parasites and is associated with various structures that have a role in cell division [5], [12]. MORN1 localizes to the centrocone, a unique structure within the nuclear envelope that houses the mitotic spindle, and to the apical and basal extremities of the IMC. MORN1 localizes most prominently to the basal complex of the IMC. This structure can be observed across the Apicomplexa and is independent of the related, yet different, division modes encountered across the Apicomplexa [12]. In the case of *Toxoplasma* cell division, the basal complex acts in place of an actin-independent contractile ring in the late stages of cell division. The contractile force is probably generated by Centrin2, a calcium dependent contractile protein [6]. In addition to MORN1 and Centrin2, several intermediate filament-like alveolin domain containing proteins (basal IMC proteins) assemble into the basal complex as well. These basal IMC proteins assemble on the cortical cytoskeleton of the early daughter buds but halfway through bud assembly transition to the basal complex. This timing coincides with the onset of basal complex contraction [4].

MORN1 is composed of a short unique N-terminus, 14 membrane occupation and recognition nexus (MORN) repeats, a linker of 5 amino acids between MORN domains 6 and 7 and a C-terminus composed of a 15^th^ but degenerated and partial MORN motif [5]. Repetitive MORN motifs in various proteins across higher eukaryotes have been implicated in various processes, including protein oligomerization [13], [14], mediating interactions with other proteins in larger protein complexes [15], [16] and membrane-membrane interactions e.g. tethering the sarcoplasmic reticulum to the plasma-membrane in cardiomyocytes [17], [18]. There is also evidence that the MORN motifs might interact directly with phospholipids to facilitate membrane association [19]. Differential detergent extractions of MORN1 from *T. gondii* and *P. falciparum* have provided evidence that MORN1 is associated with both the cytoskeleton and a membrane component, consistent with its localization at the IMC extremities [5], [12]. Overexpression of a C-terminally YFP-tagged MORN1 construct results in a dominant negative phenotype wherein the formation of the IMC is arrested shortly after the initiation of its formation. However, this defect is uncoupled from the formation of the microtubular skeleton, which remains unaffected [5]. Furthermore, upon overexpression of an N-terminal YFP fusion, MORN1 fibers emanating from the spindle pole are observed [7].

The distinct sub-cellular localization of MORN1 in the Apicomplexa and the function of MORN motifs in other systems suggest that MORN1 may act as a scaffolding protein organizing protein complexes with a critical role in mitosis and cytokinesis. To test this hypothesis we generated a conditional MORN1 knock-out in *T. gondii* and dissected its phenotype in tachyzoites. Our results show that MORN1 is critical for viability and that the lack of MORN1 results in defective organelle partitioning and basal complex assembly. The most distinctive effect of these defects is the appearance of double and multi-headed parasites as a result of incomplete daughter abscission and sequential rounds of budding. These results indicate the absence of checkpoints on daughter maturation before entering the next round of cell division. Furthermore, we exploited the conditional MORN1 knock-out parasites to identify functional domains within MORN1 by complementation with a series of MORN1 deletion constructs.

## Results

### Isolation of a conditional MORN1 knock-out parasite line

**Figure 1:**
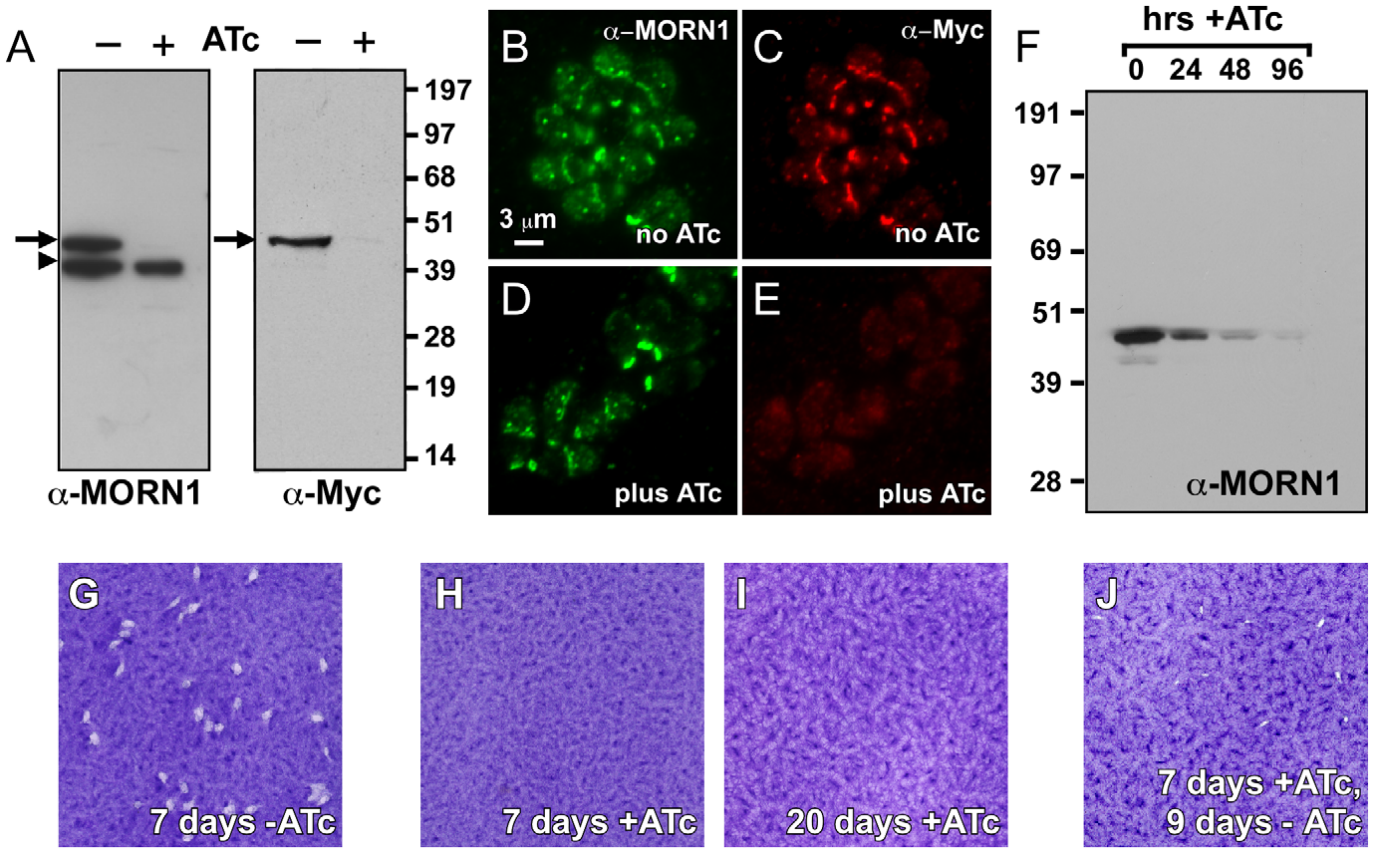
Establishment and validation of a conditional MORN1 knock-out parasite. (A–E) MORN1 expression levels assessed in pseudo-diploid parasites expressing an ATc conditional Myc_2_-tagged MORN1 allele driven by the Tet7sag1 promoter next to the endogenous MORN1 allele. (A) Total lysates harvested from uninduced (-ATc) and 24 hrs induced (+ATc) parasites were western blotted and probed with MORN1 and Myc antibodies as indicated. Arrows mark the Myc tagged MORN1; the arrowhead marks endogenous MORN1. (B–E). MORN1 (green) and Myc (red) expression under the same conditions as (A) assessed by immunofluorescence. (F) Time-course of MORN1 expression in the knock-out parasites at 0, 24, 48 and 96 hrs post ATc addition. (G–J) Plaque assays of the MORN1-KO parasites in absence of ATc (G), under ATc for 7 days (H) or 20 days (I) and for 7 days under ATc followed by 9 days without ATc (J). The absence of plaques in H and I indicates no viable parasites whereas the small plaque size and low numbers in panel J indicate aborted recovery of the phenotype. Additional recovery assays are shown in Supplementary Figure S2. A concentration of 1.0 µg/ml ATc was used throughout all experiments.

To study the function of MORN1 we constructed a null mutant in *T. gondii*. We anticipated that MORN1 likely would be essential and therefore we engineered a conditional knock-out parasite line using the Tet-off system [20], [21], [22]. We first generated a pseudo-diploid line conditionally expressing the MORN1 coding sequence fused to an N-terminal tandem Myc-tag (Myc_2_) under control of the Tet7sag1 promoter. A clone was selected wherein the expression levels of the Myc_2_-tagged transgene closely matched that of the endogenous locus (Fig. 1A). We further established by immunofluorescence and western blotting that expression of the transgene was down regulated to undetectable levels upon culture in the presence of anhydrous tetracycline (ATc) (Fig. 1B–E). Subsequently the endogenous locus was deleted by double-homologous cross-over with a selection marker. As initial plasmid-based targeting constructs had failed to yield a knock-out, we constructed a targeting cosmid by recombineering cosmid clone PSBMG48 [23]. Using the modified cosmid, the MORN1 open reading frame was replaced with the chloramphenicol acetyl transferase (CAT) gene. We successfully established a clonal line in which the endogenous locus was replaced with the CAT gene (Supplementary Figure S1; clone C9 was used for all experiments). The kinetics of repression of Myc_2_-tagged MORN1 in response to ATc addition in this line was assessed by western blotting (Fig. 1F). Expression was reduced to approximately 50% after 24 hrs in the presence of ATc and no MORN1 was detectable after 96 hrs.

The viability of the conditional MORN1-KO parasites was determined by plaque assays (Fig. 1G–J and S2). No growth was detected up to 20 days in cultures grown in the presence of ATc. Furthermore, we tested if there was any recovery after prolonged ATc exposure. We grew parasites in the presence of ATc for 7 days, followed by 9 days of growth in the absence of ATc to allow plaque formation. As shown in Figure 1J, a limited number of small plaques could be discerned. This indicates that the induced parasites are unable to fully recover since single wild-type parasites form much larger plaques after only 7 days of growth (Fig. 1G).

### MORN1-KO parasites have a defect in daughter abscission

**Figure 2:**
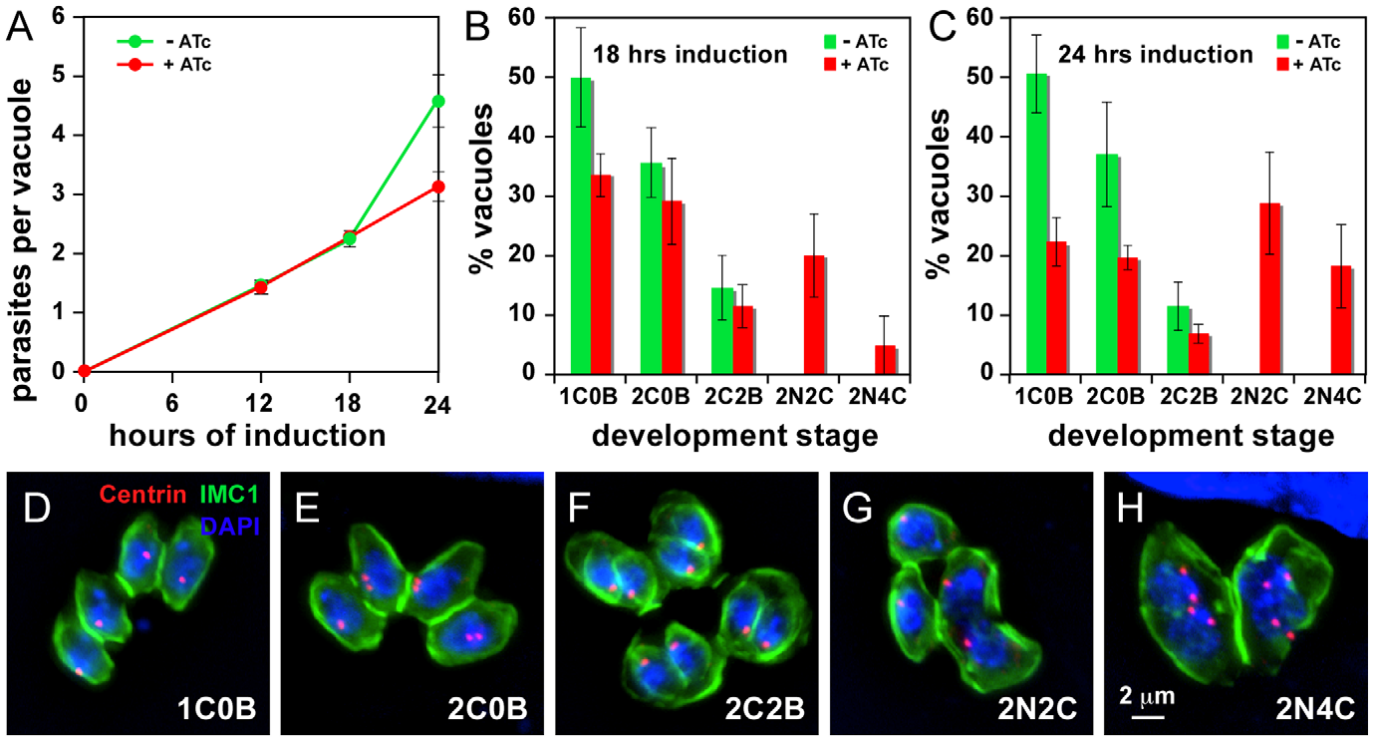
MORN1 knock-out tachyzoites display a lethal phenotype. (A) Growth curves of the conditional MORN1 knock-out parasites with and without ATc induction displaying a decreased growth rate at 24 hrs post induction. Vacuole sizes of unsynchronized parasites were scored by fluorescence microscopy on fixed parasites using IMC3 antiserum; error bars denote standard deviations of three independent experiments. (B, C) Developmental progression analysis through the tachyzoite division cycle upon 18 hrs (B) and 24 hrs (C) of ATc induction. Developmental stages were defined using centrosome and daughter budding markers as indicated in (D–H). Cultures were unsynchronized and over 100 vacuoles per condition were counted in three independent experiments; error bars denote standard deviation. (D–H) Phenotype development was assessed using centrosome (α-centrin) and cytoskeleton scaffold (α-IMC1) markers as well as DAPI for the nuclear material. Stages were defined as indicated at the bottom right; numbers indicate the number of either centrosomes “C”, daughter buds “B” or nuclei “N”. Stages 2N2C and 2N4C were only detected in the ATc induced samples. Classes shown in (G) and (H) were differentiated for presence of buds as well, but this percentage was very low (1–2%) and is not included in the graphs.

Next we wanted to determine why the MORN1-KO mutants fail to grow. As shown in Figure 2A, the mutant parasites initially grow with indistinguishable rates regardless of the presence or absence of ATc, however, treated mutants start to slow down after 18 hrs incubation in the presence of ATc, coinciding with the depletion of MORN1 (Fig. 1F). To assess whether the parasites were arresting at a specific point in their cell division cycle, we performed IFA experiments to follow their development. We used antibodies against the centrosome and the cytoskeleton (IMC1 antibody) and co-stained with DAPI to monitor nuclear development. Based on the number of centrosomes, daughter buds, and nuclei we scored parasite development into the classes shown in Figure 2D–H. In the presence of ATc we observed large parasites with two segregated nuclei in the absence of IMC daughter buds (Fig. 2G). In addition, we noted that parasites with two nuclei contained either one or two centrosomes per nucleus (Fig. 2H). Furthermore, not all parasites within a single vacuole displayed these distinct phenotypes (Fig. 2G). For counting purposes, vacuoles with at least one mutant phenotype were scored as mutant vacuoles (categories 2N2C or 2N4C; Fig. 2G,H). The incidence of the various classes was quantified and plotted for two time points in Figures 2B and C. Between 18 and 24 hrs of ATc induction the 2N2C population increases from 20% to 30% whereas the 2N4C population increases from 5% to 30%. These data suggest that parasites first display the 2N2C phenotype and then divide their centrosomes and develop into the 2N4C phenotype. This indicates that the nuclear development cycle continues in these mutants.

**Figure 3:**
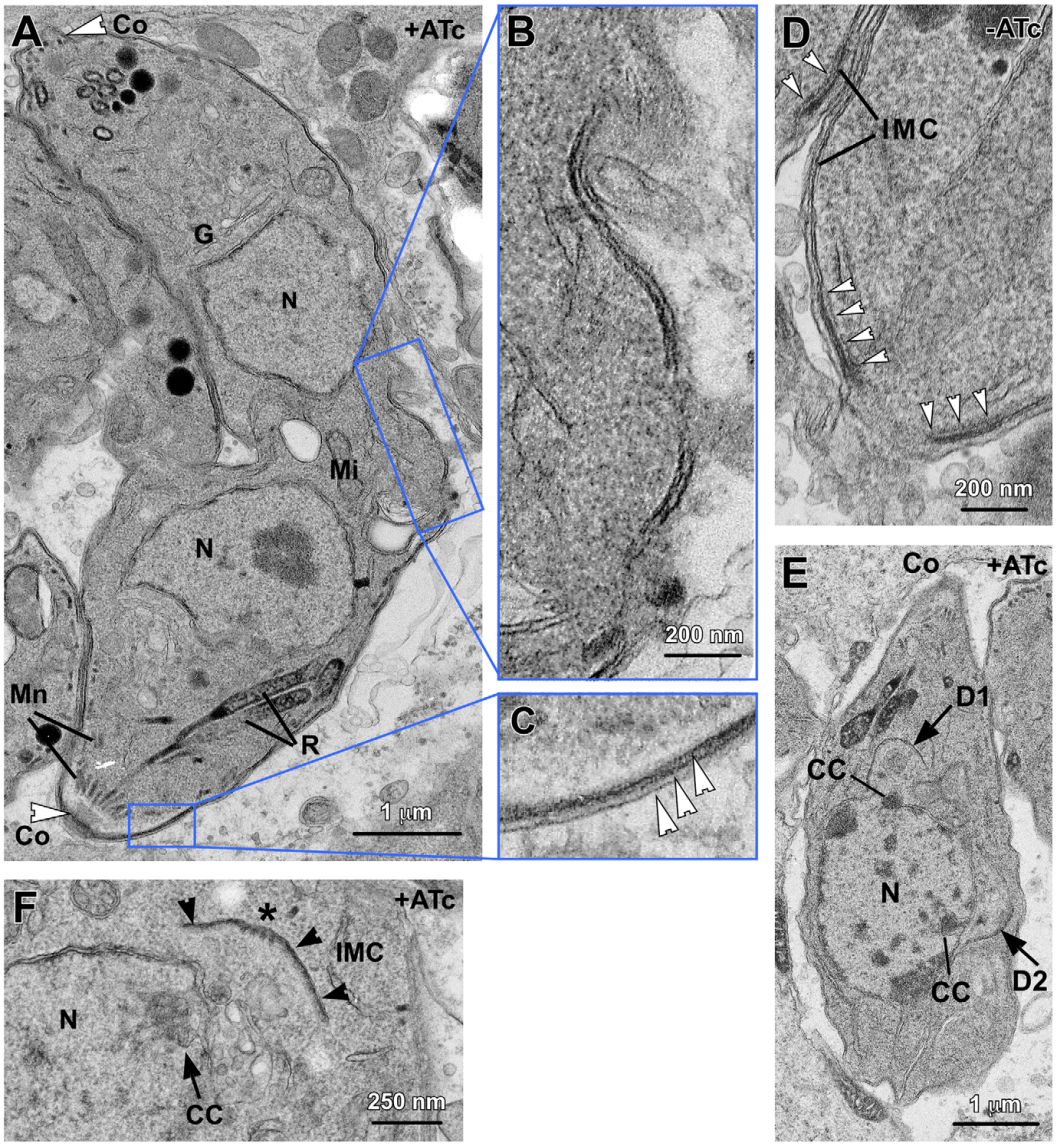
MORN1-KO mutants display a multi-headed, division phenotype. (A–F) DNA content was assessed by flow cytometry with or without ATc for time periods as indicated. Defined populations representing 1N, 1.8N, 2.7N and 3.5N nuclear contents could be differentiated (B–D) and some are quantified in (E). Extracellular parasites lysed out after 48 hrs harbor large populations of 1.8N, and 3.5N nuclear content. (G) Extracellular parasites lysed out after 48 hrs were examined by immunofluorescence and identified multi-headed parasites with multiple nuclei (DAPI), mature subpellicular microtubules (α-tubulin) and IMC filament cytoskeleton (α-IMC3). (H) Single fluorescence channels and phase-contrast image of panel (G). (I) Extracellular parasites lysed out after 48 hrs were examined by scanning electron microscopy which identified basally conjoined parasites with 2, 4, 8 or 16 apical ends as indicated.

Since centrosome duplication coincides with entry into S-phase, we wanted to assess whether the loss of MORN1 would lead to an increase in DNA content. We measured parasite DNA content by flow cytometry at different times after mutant induction with ATc (Fig. 3). In the absence of ATc we observe the typical cell cycle distribution previously reported [24]: ∼60% of the parasites are haploid and in G1 while 20–25% show a 1.8N DNA content (Fig. 3A). In addition we observed a small, unusual population with an approximately 2.7N DNA content. This most likely originates in two parasites sticking together (e.g. 1N+1.8N parasite). The artificial assignment of this population is strengthened by its constant size across the time points in the presence of ATc, whereas all other populations shift under these conditions (Fig. 3E). Usually this artificial population is excluded from cytometry analysis by gating on the single-cell population. However, for this analysis we did not implement such tight gates in order to facilitate the detection of the larger bi-nucleated parasites as observed in Figure 2G and H. With the same goal in mind, we filtered the parasites over a 12 µm pore size filter instead of the regular 3 µm pore filter. Upon addition of ATc a new population appears with 3.5N DNA content, the size of which increases with prolonged ATc exposure (Fig. 3B–E). Moreover, in egressed, extracellular parasites collected from the culture supernatant after 48 hrs of ATc induction the 3.5N category represents 19% of the total population (Fig. 3F). The 3.5N population most likely reflects entry into another round of DNA synthesis of the double-headed bi-nucleate parasites as this number closely matches 2*1.8N, which is the natural arrest before undergoing mitosis [24]. Together with the duplication of the centrosomes, this indicates that the nuclear cycle indeed progresses in absence of MORN1.

We next determined whether the egressed population after 48 hrs is indeed enriched in double-headed phenotypes. We harvested these parasites without filtration and performed immunofluorescence microscopy. This confirmed the presence of an increased number of double-headed parasites. Moreover, we identified monstrous multi-headed parasites containing multiple nuclei conjoined by a central mass (Fig. 3G,H). Interestingly, all the heads contained an intact microtubular and IMC-filament cytoskeleton. Because the IFA data was hard to interpret due to the presence of the multiple heads lying on top of each other we studied the appearance of these parasites by scanning electron microscopy. As shown in Figure 3I we observed double-headed, 4-headed, 8-headed and even 16-headed parasites conjoined at the basal end. The 4-headed parasites were the most abundant. Taken together, these data suggest that the lack of MORN1 results in incomplete cytokinesis, in particular incomplete abscission. However, this does not prevent the initiation of new rounds of DNA replication and budding and results in the formation of multi-headed parasites.

### Ultrastructural analysis of the MORN1 null mutant

**Figure 4:**
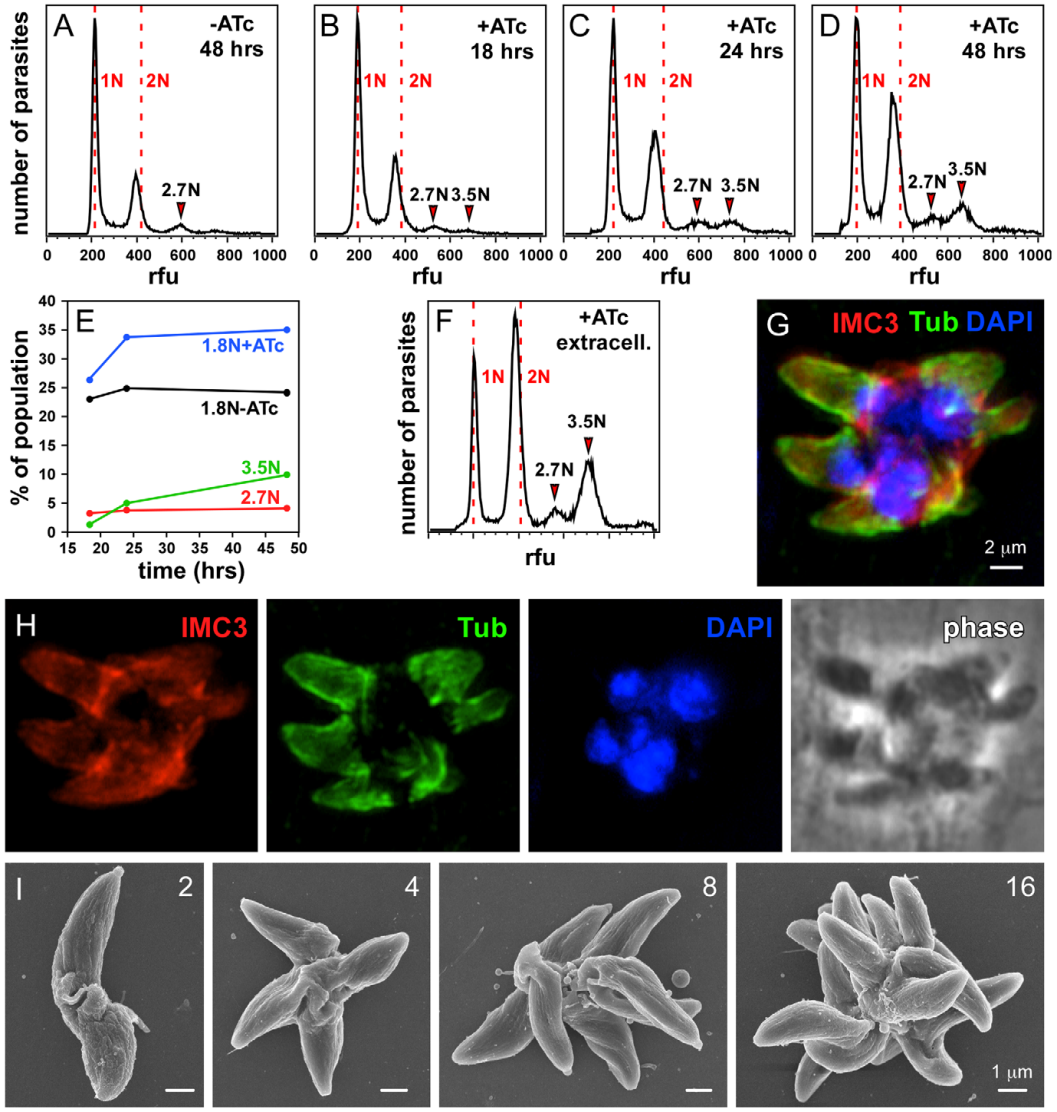
Late stages in daughter separation are affected in the MORN1-KO mutants. MORN1-KO parasites were induced for 24 hrs with ATc. (A) A double-headed MORN1-KO parasite displaying two intact conoids (Co, arrowheads), normal appearing micronemes (Mn), rhoptries (R), Golgi (G), mitochondrion (Mi) and two nuclei (N). (B) Enlargement of the boxed area in panel A shows discontinuous IMC at the central area where the daughters are connected. (C) Enlargement of the boxed area in panel A shows the three layers of the pellicle as marked by arrowheads (plasma membrane, outer IMC membrane, inner IMC membrane). (D) The basal end of MORN1-KO parasites grown in the absence of ATc displays electron dense matter at the end of the IMC (arrowheads) known as the basal complex [4], which is not observed in the double-headed parasites. (E) Early daughter buds after completion of mitosis. Centrocones (spindle poles) are marked by CC; N indicates the nucleus, Co indicates the mother parasite's conoid, D1 and D2 mark the two daughter buds with arrows. (F) Another dividing MORN1-KO parasite at higher magnification showing a normal appearing centrocone (CC, arrow) and early forming daughter parasite. The IMC is marked with arrowheads; the asterisk marks the location of four microtubules under the central part of the bud, indicating that early daughter formation is normal. Note the presence of microtubules running through the centrocone into the nucleoplasm.

To examine the development of the MORN1 null phenotype in more detail we performed transmission electron microscopy (Fig. 4). The double-headed parasite in Figure 4A clearly shows that the daughters are conjoined at their basal ends since two mature apical complexes are present on opposing sites. The appearance and localization of conoids and apical secretory organelles (micronemes and rhoptries) is normal. Two nuclei are present whereas the Golgi apparatus and mitochondria conform to the localizations expected based on their normal division and partitioning dynamics; the Golgi is localized apical to the nucleus and the mitochondrion between the nuclei reflecting the late entry of this organelle into the daughter buds [25]. No remnant of the mother parasite can be discerned, which indicates that the failure of separation occurs late in cell division. The connection between the conjoined daughters is very wide and unconstricted, suggesting a defect in the contraction of the basal complex. Comparison of the cytoskeleton and plasma membrane where the two parasites are conjoined (Fig. 4B) with the wild-type basal complex morphology (Fig. 4D) revealed that the electron dense area representing the basal complex is missing at the end of the IMC of the conjoined parasites. Furthermore, the IMC In the conjoined area appears to have several gaps, suggesting a potentially jagged basal IMC ending or this could hint at a few minor remnants of the mother IMC. However, “gapped” IMC is not the typical appearance observed when the mother disassembles and the daughters emerge, but are closely apposed structures rather than gapped [9], [11]. A close-up of the parasite's periphery at a more apical location shows that no mother IMC is present since the normal, three-membrane layered pellicle composed of plasma membrane and the two IMC membranes can clearly be discerned. This also indicates that the emerging daughters have a mature pellicle (Fig. 4C).

Since absence of MORN1 from the centrocone has the potential to result in a defect in mitosis, even if DNA synthesis is not affected, we assessed the appearance of the centrocone in more detail as well. Figure 4E and F show that mitosis progressed normally after 24 hrs ATc induction and that the centrocones appear as the typical electron dense nuclear envelope invaginations with microtubules running through the centrocone into the nucleoplasm [5]. Furthermore, early daughter formation displays all the normal features, including several microtubules underlying the early IMC (Fig. 4F) [2], [26].

### Assembly and function of the basal complex is impaired in the MORN1-KO

**Figure 5:**
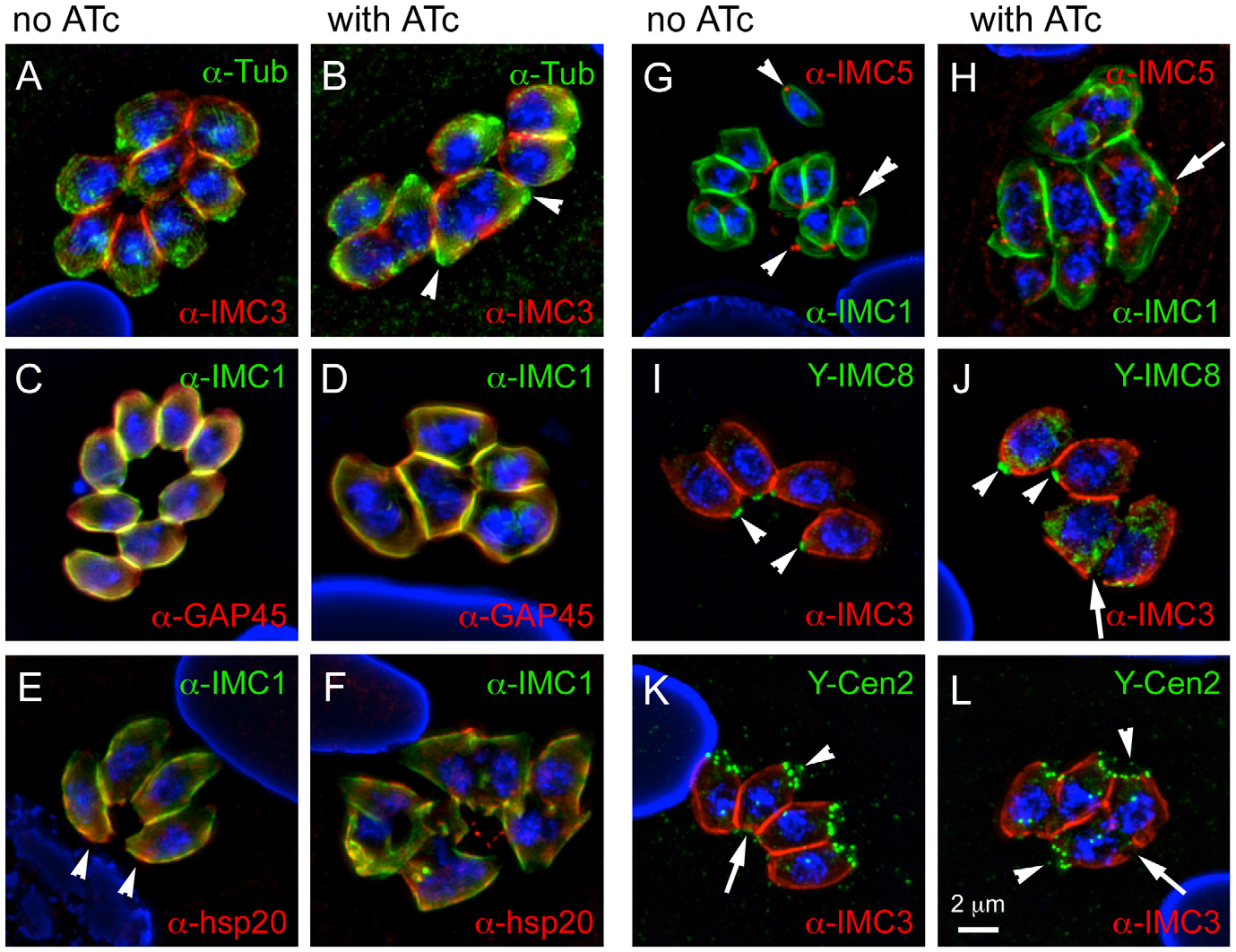
MORN1-KO parasites are defective in basal complex assembly. MORN1-KO parasites were grown for 24 hrs in the absence (A,C,E,G,I,K) or presence (B,D,F,H,J,L) of ATc and subjected to immunofluorescence using either IMC1 or IMC3 antiserum, to highlight the IMC, in combination with the following antibody markers: (A,B) α-tubulin antibody, to highlight the subpellicular microtubular cytoskeleton; (C,D) GAP45 as a marker for maturation of the pellicle; (E,F) hsp20 as an independent IMC marker for pellicle maturation; (G,H) IMC5 antibody and (I,J) DD-YFP-IMC8 (Y-IMC8) as markers for the basal complex; (K,L) DD-YFP-Centrin2 (Y-Cen2) as a marker for the apical end and the basal complex. In all cases nuclear material was stained with DAPI. 1 µM Shield1 was added for 24 hrs to stabilize the DD domain fusion proteins. DD-tags were stained with α-FKBP12 (green). (A) Arrowheads indicate the two conoids present in the single cytoplasmic mass of a double-headed parasite. (E) Arrowheads indicate the basal accumulation of hsp20 in some uninduced parasites. (G-J) Arrowheads indicate the basal complex in some mother parasites, the double arrowhead indicates the contracting basal complex in a budding daughter, arrow indicates where the basal complex should have been assembled. (K,L) Arrowheads indicate the conoid at the apical end of some parasites whereas arrows indicate the basal complex (left panel), or where the basal complex should have been assembled (right panel). Single channel fluorescence panels are available in Supplementary Figure S3.

To pinpoint the defect in daughter abscission at the molecular level we evaluated the development of the cytoskeleton in the MORN1 mutant using a battery of markers to highlight different aspects of the budding, maturing and mature cytoskeleton (Fig. 5). We first analyzed the tubulin cytoskeleton using an α-tubulin antibody. Under MORN1-KO conditions we observed parasites with two conoids without a basal end (Fig. 5B). This suggested that the daughters had fully matured and that in turn the structures of the mother cell had been disassembled as we also observed by TEM (Fig. 4A,C). To confirm this we tested if the IMC of the daughter parasites was attached to the plasma membrane. This association represents the final step of maturation of the daughter pellicle for which GAP45 has been established as a marker [11]. GAP45 anchors the gliding machinery in the IMC and is only inserted into the IMC upon association of the IMC with the plasma membrane. Co-staining for GAP45 and IMC1 revealed perfect co-localization (Fig. 5D) further supporting daughter maturation and mother disassembly in the mutant. We used an additional marker for maturation, hsp20, which starts associating with the daughter buds in the second half of division, after the bud reaches its widest point [27]. This maturation step precedes the insertion of GAP45. Fitting this pattern, hsp20 was observed in association with the pellicle of the double-headed daughters (Fig. 5F).

MORN1 mutant parasites assemble fully mature daughters yet fail to complete cytokinesis at their basal ends, which is likely due to the absence of the basal complex (Fig. 4A,B). Since MORN1 is a major component of the basal complex this suggests that the absence of MORN1 could result in an incomplete or at least a non-functional basal complex, potentially missing other components. Besides MORN1, the basal complex has been shown to contain several other parts, including Centrin2 and a dynein light chain (TgDLC) [7]. In addition, we recently identified several intermediate filament-like proteins residing in the basal complex, IMC5, IMC8, IMC9 and IMC13 [4]. These basal IMC proteins initially assemble on the cortical cytoskeleton of early daughter buds. When the bud reaches its widest point, the basal IMC proteins shift from their initial localization toward the posterior end and become part of the basal complex where they remain in mature parasites. The timing of this shift coincides with the recruitment of Centrin2 and these events mark the start of constriction of the basal complex to result in a cytoskeleton tapering toward the basal end [6]. Due to the spatio-temporal behavior of the basal IMC proteins they therefore provide good markers to study to what degree the basal complex is assembled in the MORN1-KO mutant. Basal IMC proteins IMC5 and IMC8 were not observed in the central region of the double-headed parasites where the basal complexes would be expected (Fig. 5H,J). Instead of the typical ring-shaped basal complex, IMC5 and IMC8 are observed as punctuate structures, often around the widest section of the double-headed parasites. Comparable data were obtained for IMC9 and IMC5, the latter using antibodies and YFP fusions, and both displayed similar localization patterns (Fig. 5H,J and Fig. S3M-P). We were unable to assess the localization of IMC13 in the MORN1-KO background since expression of YFP-tagged IMC13 was only achieved with the Tet-on system [4], which is not compatible with the Tet-off system controlling Myc_2_-MORN1. The mother's basal complex is the last structure to be disassembled in the cytokinetic process, for which the basal IMC proteins can serve as markers as well [4]. In the induced parasites we never observe parasites with a normal morphology, specifically, tapered basal ends, wherein we can not detect the basal IMC components. Since in none of double-headed parasites we observe remnants of the mother's basal complex, this provides further evidence that disassembly of the mother is completed.

Besides the basal IMC proteins we addressed whether Centrin2 assembled in the basal complex of the mutants. In wild-type parasites Centrin2 has multiple locations, with the basal complex actually being the most modest signal [6], [7]. The more prominent localization of Centrin2 is at the very apical tip of the apical complex as well as a ring of six annuli on the border of the apical cap region. Therefore Centrin2 also provides a good marker for different features at the apical end of the parasites. In the double-headed MORN1 mutants we detected the apical Centrin2 structures on both ends of the parasite (Fig. 5L). This further supports the maturated nature of the apical cytoskeleton structures of the mutant parasites. At the basal complex, we could not detect any Centrin2. Moreover, TgDLC could also not be detected in basal complex in the absence of MORN1 [28]. Therefore, none of the known basal complex components appear to assemble in the absence of MORN1. Taken together, this data supports the evidence provided by the TEM that the entire basal complex is missing and suggests that assembly of the basal complex is critically dependent on the presence of MORN1. Consequently, the absence of the basal complex prevents the contraction of the basal end of the cytoskeleton in the late steps of cytokinesis.

### MORN1-KO parasites display impaired apicoplast segregation

The internally assembling cytoskeleton driving *Toxoplasma* cell division serves as a scaffold for the assembly of new secretory organelles such as micronemes and rhoptries while other organelles such as the single copy Golgi, apicoplast and mitochondrion divide and partition in association with the division machinery [25]. To address whether the division of any of these organelles was affected in the MORN1 null phenotype we used a series of organelle specific markers. An organelle for which division has been suggested to be mediated at least in part by the MORN1-containing basal complex is the apicoplast [29].

**Figure 6:**
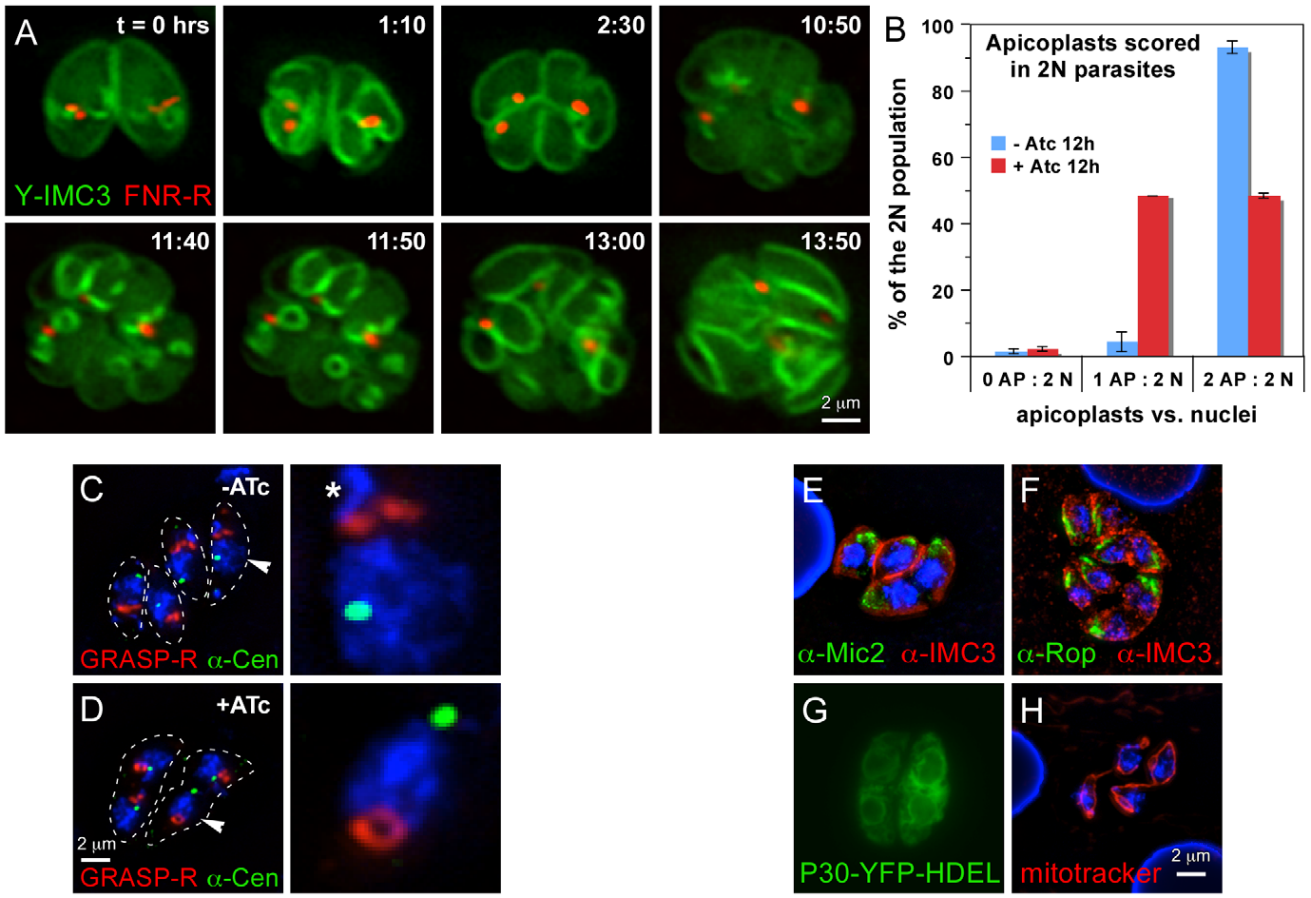
MORN1-KO parasites display a defect in apicoplast segregation and Golgi apparatus development. (A) Selected panels of a time-lapse movie S1 of apicoplast division and parasite development in presence of ATc. ATc was added 12 hrs before t = 0 hrs. MORN1-KO parasites are expressing YFP-IMC3 and FNR-RFP marking the cortical cytoskeleton and the apicoplast, respectively. (B) The incidence of plastid loss after 12 hrs of ATc induction quantified in parasites with two nuclei (2N). Three categories are discerned: no plastid (0 AP), 1 plastid (1 AP) or 2 plastids (2 AP). (C,D) MORN1-KO parasites expressing the Golgi apparatus marker GRASP55-RFP, co-stained with DAPI and centrin antiserum. The parasites are outlined by a dotted line. In the right panels the nucleus plus Golgi and centrosome are 400% enlarged of the nuclei marked with an arrowhead in the left panel. Asterisk marks the nuclear content of the apicoplast. (E–H) Markers for the micronemes (α-Mic2), rhoptries (α-Rop), the endoplasmic reticulum (P30-YFP-HDEL) or the mitochondria (mitotracker) in the induced MORN1-KO parasites indicate normal development and segregation of these organelles.

The apicoplast is a unique plastid-like organelle derived from a red alga by secondary endosymbiosis [30]. The apicoplast shows distinct differences to typical chloroplasts found in plants including its mechanism of replication, which is linked to the centrosomes of the mitotic spindle [31], [32]. A recent model suggested that fission of the organelle occurs in a two-step process: constriction of the apicoplast by the basal complex followed by abscission using the dynamin-like protein DrpA [29]. To directly test this idea using the MORN1 mutant we observed plastid morphology upon ATc treatment by immunofluorescence (Fig. 6). We note that loss of MORN1 has immediate and profound effects on apicoplast fission leading to daughter cells lacking the organelle. We quantified this effect after 12 hrs of ATc induction by staining apicoplasts with an antibody for *T. gondii* Cpn60 [33] and nuclei with DAPI. In wild type parasites nuclear division coincides with apicoplast fission [29], [31]. In the absence of ATc we observe two daughter apicoplasts in 93.5% of the parasites in which nuclear division has occurred (Fig. 6B, n = 100). In contrast, after 12 hours of exposure to ATc only 50% of parasites with two nuclei also show two plastids. To characterize this phenotype in a dynamic fashion we introduced two fluorescent marker proteins into the MORN1 mutant, FNR-RFP to label the apicoplast and YFP-IMC3 to visualize budding daughters. We performed *in vivo* time-lapse imaging experiments to follow apicoplast replication in the mutant. Frames highlighting key events from a representative movie are shown in Figure 6A and the full movie is provided as Supplementary Movie S1. In the two parasites shown the apicoplast is inserted into the daughter buds and elongates as previously described [31]. While fission proceeded in the left parasite it failed in the right cell leading to unequal segregation. The cells were imaged through a second round of attempted cell division and continued MORN1 depletion. After 24 hours of ATc treatment a complete loss of apicoplast fission is observed in all cells. We note that daughter buds are still formed yet fail to constrict.

Among the other organelles studied, the Golgi apparatus displayed an architectural change in the MORN1 null phenotype. The Golgi is first organelle that divides in the *Toxoplasma* division cycle by going through an extension in the second half of G1 followed by a pause while the centrosomes are dividing before the actual fission of the organelle at the boundary of G1 and S-phase [25], [34], [35]. Using GRASP55-RFP transgenics, we noticed that the appearance of the Golgi in MORN1 null parasites was often times extended, bi-lobed, or appeared as a donut-shape (Fig. 6C,D). Such appearance is consistent with a dividing Golgi apparatus, although the donut shape is quite distinct and unique to the MORN1-KO phenotype. We never observed parasites missing a Golgi apparatus suggesting that division and partitioning were not affected by the distinct morphology.

Other organelles we studied did not display any defects in assembly and/or partitioning. The micronemes and rhoptries were observed at both apical ends of the double-headed parasites showing their organellogenesis proceeds normally (Fig. 6E,F). Considering the dividing organelles, we evaluated the ER (Fig. 6G) and mitochondria (Fig. 6H). Both these organelles co-segregate with the nuclei in normal cell division and are the last organelles to enter the daughter parasites [25]. In the double-headed parasites the ER and the mitochondrion do co-segregate with the nuclei, but segregation is not 100% complete. Considering the lack of abscission and the late timing for segregation of these organelles, this is consistent with a defect in segregating of the ER and the mitochondrion.

### Full-length MORN1 is required for functional rescue of the knock-out

The conditional knock-out MORN1 parasite provides a powerful tool to identify functional domains within MORN1 through complementation studies with mutated MORN1 constructs. For example this could address whether different sections of MORN1 are required for centrocone localization than for apical or basal complex localization. In addition, we also wished to assure that the MORN1 null phenotype is indeed due to lack of MORN1. To answer these questions we generated a full-length MORN1 complementation construct as well as a series of MORN1 deletion mutants and a point mutant. To guide the construction of the MORN deletion mutants we assessed MORN1 for any functional motifs. As reported before, the MORN1 protein consists of 14 complete MORN domains and 1 partial MORN domain (PM) at the C-terminus (Fig. S4; [5]). The N- and C-terminus both have short sections not part of a MORN domain whereas a short 5 amino acid linker between MORN domains 6 and 7 is present. A previously unrecognized potentially functional feature in MORN1 is a cysteine residue at position 4, which is a predicted palmitoylation site (the suite available from the Institute of Molecular Pathology Bioinformatics Group (http://mendel.imp.ac.at/mendeljsp/index.jsp) was used; no additional acylation sites were detected) [36], [37]. Palmitoylation of MORN1 would provide a model for the membrane association of MORN1 observed by differential detergent extraction [5], which could potentially anchor MORN1 in the nuclear envelope and/or the IMC membrane.

To dissect the highly modular MORN1 structure we designed a ‘sliding window’ of MORN domain deletion mutants and additionally generated a mutant construct wherein the linker was deleted (MORN1.DEL; see Fig. S4A for an overview of constructs). To address the relevance of the predicted palmitoylation site we generated a full-length MORN1 construct wherein the Cys at position 4 is changed into an Ala residue (MORN1.C4A). To streamline the MORN1 null mutant complementation studies, we first assessed localization of these constructs in the wild type background to determine which regions contained specific localization information and how localization relates with the number of MORN domains (Fig. S4). These experiments showed that wild-type MORN1 localization is observed with either the N- or C-terminal half of MORN1 (split at the linker region). In addition this showed that constructs with less than 4 MORN domains localize to the cytoplasm. Furthermore, the general observation was that constructs spanning the 5 amino acid linker between MORN induce a strong aggregation of the YFP fusion protein, which in many cases was lethal (e.g. as observed when full-length MORN1-YFP is overexpressed [5]). Because these experiments were performed in endogenous MORN1 expressing parasites, some of the localizations observed for the YFP-tagged deletion constructs could be due to association with the full-length version.

**Figure 7:**
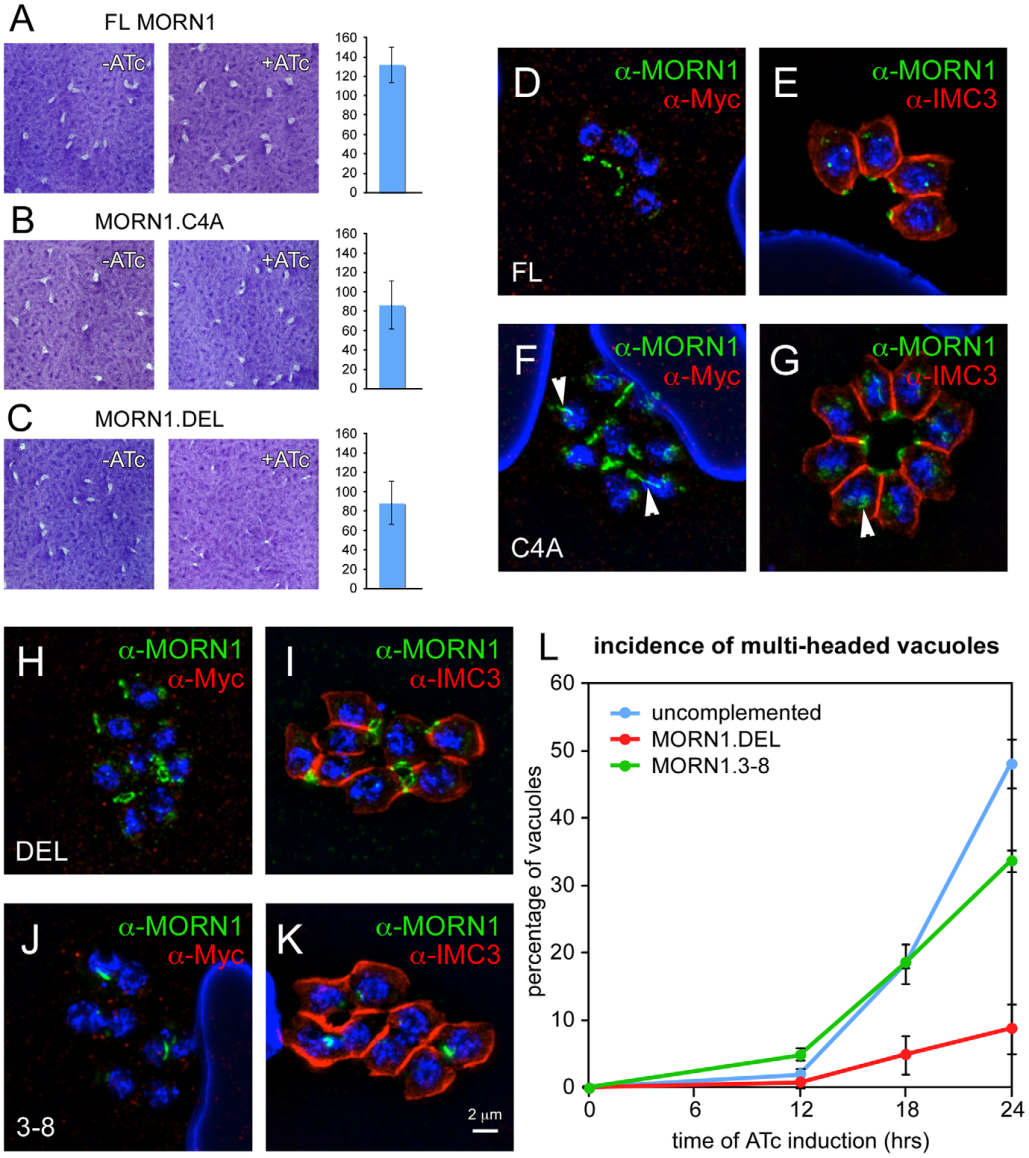
Complementation of the MORN1-KO parasites with various MORN1 constructs. (A–C) Plaque assays of MORN1-KO parasites complemented with full length (FL) MORN1, full length MORN1 with a Cys 4 to Ala point mutation (MORN1.C4A) to disrupt a predicted palmitoylation site and MORN1 wherein the linker region was removed (MORN1.DEL). The graphs on the right display the number of plaques relative to the control without ATc (percentage). Average of three independent experiments; error bars denote standard deviation. (D–K) MORN1-KO parasites complemented with full-length MORN1 (D,E), MORN1.C4A (F,G), MORN1.DEL (H,I) or MORN1.3–8 (J,K) stained with α-Myc and α-MORN1 (D,F,H,J) or α-MORN1 with α-IMC3 (E,G,I,K). Arrowheads in (F,G) point out the fiber emanating from the centrocone in some of the MORN1.C4A complemented parasites. (L). Quantification of the incidence of double-headed parasites in the MORN1 null phenotype (uncomplemented), the MORN1-KO complemented with the MORN1.DEL or the MORN1.3–8 construct plotted as a function of time of ATc induction. At least 150 vacuoles were scored at each condition. Average of three independent experiments is shown; error bars denote standard deviation. Single channel fluorescence figures for panels (D–K) are available in Supplementary Figure S5.

Based on the above described localization results we selected a subset of constructs spanning the range of observed localization patterns for complementation of the MORN1 null mutants (see Fig. S4 for selected constructs). All constructs were untagged and driven by the endogenous MORN1 promoter. Stable transfectants with these constructs were generated using the BLE selection marker. Initially we attempted functional complementation by selecting for viable parasites under ATc pressure alone. However, this led to loss of the down-regulation feature of the conditional allele upon ATc addition. This most likely is the consequence of chromosomal re-arrangements in the surviving populations. Complementation of the conditional MORN1 knock-out line with a construct encoding full length MORN1 resulted in complete growth restoration as assessed by plaque assays (Fig. 7A). In addition, the sub cellular localization of complemented MORN1 was similar to wild-type MORN1 (Fig. 7D,E). Complementing MORN1 was differentiated from the conditional Myc_2_-tagged MORN1 by co-staining with Myc and MORN1 antibodies (Fig. 7D). This demonstrates that the defect observed in the conditional MORN1-KO parasite is indeed due to loss of MORN1 alone.

Subsequently we tested whether the MORN1.C4A palmitoylation site mutant was able to rescue the phenotype. This mutant allele provided full restoration of the growth defect as assessed by plaque assays (Fig. 7B). However, localization of MORN1.C4A differed from wild-type MORN1 at the centrocone whereas localization at the basal end was normal (Fig. 7F,G). A fiber emanating from the centrocone is observed for MORN1.C4A. As such, if MORN1 is palmitoylated, it plays a potential role at the centrocone but not in the localization to the basal complex.

The only other MORN1 construct that was able to restore growth of the MORN1 null mutant was the allele with the linker deletion (MORN1.DEL). Growth restoration was not to full wild-type levels as judged by the size of the plaques, although no significant reduction in plaque number was observed (Fig. 7C). The cause of reduced plaque size appears to originate in the presence of the double-headed phenotype in some of the MORN1.DEL complemented parasites (Fig. 7H,I). To assess whether the incidence of the multi-headed phenotype was indeed lower than in the uncomplemented mutants we counted the number of double-headed parasites at different time-points of ATc induction (Fig. 7L). Whereas the incidence of the double-headed phenotype in the uncomplemented MORN1-KO reaches 18% and 48% after 18 and 24 hrs ATc induction, respectively, the incidence is only 4.8% and 8.7% for the MORN1.DEL complemented parasites at these time points, respectively. This indicates that the linker region between MORN domains 6 and 7 is critical for correct functioning of MORN1.

Complementation with the deletion constructs missing one or more MORN domains never resulted in the establishment of stable parasite populations, with the exception of a construct consisting of MORN domains 3–8 (MORN1.3–8). As shown in Figure 7L, the incidence of the double-headed phenotype closely resembled the uncomplemented mutant. The localization of MORN1.3–8 appeared as an accumulation either at or near the centrocone or between the divided nuclei; at no point was it observed associated with the cytoskeleton (Fig. 7J,K and S5). However, we noted that the Myc_2_-tagged MORN1 in these parasites did not completely disappear. This suggests that MORN1.3–8 prevented Myc_2_-MORN1 degradation and indicates the presence of strong aggregates.

## Discussion

*Toxoplasma* daughter budding is a highly complex process that requires the coordinated assembly of numerous elements. We have recently demonstrated that the cytoskeleton that organizes the IMC is of even greater complexity than previously anticipated [4]. We identified a family of 14 alveolin repeat intermediate filament-like proteins (IMC proteins) that showed a remarkably organized spatial and temporal assembly pattern for different family members. Our detailed analysis of the MORN1 null mutants strongly suggests that MORN1 is not required for initial bud formation. However, in the absence of MORN1 we do not detect any deposition of known components of the basal complex, which includes a subset of the IMC family residing in the basal complex (Figs. 4, 5, and S3). The lack of the basal complex results in a failure to contract the basal end to taper the growing cytoskeleton as part of its maturation process. This provides direct evidence that the basal complex is involved in this constriction, with Centrin2 being the most likely candidate to provide the contractile force [6]. As a result, daughter parasites are not separated from each other and form double-headed parasites with two apical ends. Remarkably, this does not prevent these Janus-headed parasites to initiate a next round of division, which is reflected in the duplication of their centrosomes (Fig. 2H) and the appearance of a population with a 3.5N nuclear content (Fig. 3). Such nuclear contents most likely represent parasites carrying two nuclei in the 1.8N arrest stage, which reflects a normal stage in the parasite's development cycle before undergoing cytokinesis [24]. Interestingly, the parasites then can undergo additional rounds of budding, but again fail to constrict their basal complex. As a result, 4-headed parasites, and upon subsequent division rounds even 8- and 16-headed parasites appear (Fig. 3G-I).

It is somewhat surprising there is no checkpoint for assembly or contraction of the basal complex before progressing toward disassembly of the mother's cytoskeleton, as this appears to be crucial for the formation of viable parasites. Therefore we questioned how this compares to well-studied model organisms. The late stages in *Toxoplasma* division, i.e. maturation of the pellicle coordinated with mother disassembly is akin to the late stages in mammalian cells completing abscission. Although in mammalian cells the initial stages of cytokineses are mediated by an actin-myosin mediated cleavage furrow ingression, which compares with the basal complex constriction in *Toxoplasma*, the final segregation is organized by a septin and membrane remodeling based machinery [38]. Mutants in the abscission machinery do lead to conjoined mammalian cells [39]. The lack of a checkpoint late in cytokinesis therefore is in line with the understanding of other systems. It is currently unclear whether *T. gondii* harbors machinery analogous to the septin based machinery to complete cell division. An argument against such machinery is that even in normal parasite development abscission is not complete and parasites are often observed to share a cytoplasmic bridge at their basal end [12]. Therefore it is more likely that *Toxoplasma* breaks the small cytoplasmic bridge present after completion of cytokinesis either by mechanical stresses within the vacuole or alternatively, by pulling away in opposite directions upon egress. Such a mechanic abscission model has been shown for the related ciliates [40]. However, in the multi-headed parasites the cytoplasmic bridge is too large to be disrupted by mechanical force.

While the effects of the loss of MORN1 on completion of cytokinesis are very strong, the formation and partitioning of other organelles and structures are largely intact with one prominent exception: the loss of apicoplast division (Fig. 6). The apicoplast is unique among plastids in that its division is linked to nuclear division by association with the centrosome [29], [31], [41]. While this finding provided a strong model for segregation it did not explain organelle fission. Some authors argued for fission rings similar to those found in the chloroplast [42], [43], however, no genes encoding homologs to the central component of that machinery FtsZ have been identified in Apicomplexa [32], [44]. Other authors suggested that cytokinesis alone and more specifically the MORN1 ring might be sufficient for apicoplast fission [31], [32]. Recent studies on the dynamin-related protein DrpA have demonstrated an absolute requirement for this protein for fission to occur. Detailed analysis of DrpA mutants showed that the MORN1 ring constricts the apicoplast during budding, but that this constriction is not sufficient for fission [29]. Using the conditional MORN1 null mutant here we demonstrate that, while not sufficient, MORN1 is clearly required for apicoplast fission. Loss of MORN1 results in loss of apicoplast fission and this effect is manifest as early as 12 hours into ATc treatment. Why two constrictive machines? Biochemical work on the role of dynamin-like proteins involved in mitochondrial division suggests that these proteins preferentially assemble in regions of initial constriction [45], [46]. We propose a model in which the MORN1 ring provides this initial constriction and thus targets DrpA to the correct site. Activity of DrpA then completes fission. This model is fully consistent with the genetic requirement of both MORN1 and DrpA.

Upon complementation of the MORN1-KO with a full-length MORN1 encoding plasmid complete restoration of the wild-type phenotype is accomplished (Fig. 7). Efforts to complement with versions of MORN1 missing one or more MORN domains failed, indicating that all MORN domains are required for function. However, complementation could be established with a MORN1 mutant carrying a point mutation in a predicted palmitoylation site (Fig. 7B,F,G). In this complementation MORN1.C4A was observed to polymerize into fibers from the centrocone. These data suggest that palmitoylation potentially plays a role in the centrocone, but not in basal complex assembly and function. Since formal proof of MORN1 palmitoylation is currently not available we cannot exclude that the amino acid change itself resulted in this effect. Furthermore, partial complementation was achieved with the construct missing the short linker between MORN domains 6 and 7 (Fig. 7C). As shown in Figure 7H and 7I, contraction of the basal complex in this mutant was of lower efficiency. This suggests that this region could act as a potential flexible hinge required for remodeling of the MORN1 complex during basal complex contraction. Fitting a critical role for this linker region, expression of MORN1.DEL in a wild-type parasite background resulted in severe aggregate formation and was lethal, possibly through a mechanism trapping endogenous MORN1 in aggregates as observed with overexpression of full-length MORN1 (Fig. S4) [5].

A MORN1 null mutant recently isolated by Heaslip and colleagues shows a similar phenotype yet is viable *in vitro* [28]. This contrasts with the loss of viability we observed for the conditional MORN1 null phenotype (Fig. 1 and S2). In part this could be the result of the strategy employed by Heaslip et al to generate an unconditional MORN1 null mutant, which inherently selected for surviving parasites in the absence of a conditional allele. In this process there is a potential selective pressure for secondary mutations suppressing the most severe manifestation of the phenotype. This interpretation is strengthened by our observation in the complementation studies; when we performed phenotypic selection under ATc only, we obtained viable parasites for most deletion constructs. However, the inducible expression of the Myc_2_-tagged MORN1 allele was lost and Myc_2_-MORN was constitutively expressed. This indicates a genomic rearrangement or other mutations accrued in this phenotypic selection process.

In conclusion, we have shown that loss of MORN1 results in an early loss of apicoplast division and prevents the assembly of the basal complex. As a result parasites fail to fully separate while still re-initiating new rounds of division resulting in the formation of the striking multi-headed parasites. Finally, we showed that the linker region between MORN domains 6 and 7 potentially serves as hinge critical for flexibility in the MORN1 complex and is a critical region for correct functioning of MORN1. Future work will be needed to address a potential role of palmitoylation and/or other post-translational modifications in the function of MORN1.

## Materials and Methods

### Parasites

RH strain parasites and transgenic derivatives were used throughout this study. Parasites were maintained in human foreskin fibroblasts (HFF) as previously described [47]. Throughout the paper a concentration of 1.0 µg/ml anhydrous tetracycline (ATc) was used to induce the knock-out phenotype.

### Plasmids

All oligonucleotides used are provided in Supplementary Table S1. To generate plasmid pTet7sag1Myc_2_-MORN1/DHFR, primers MycF-1, MycF-2, MycR-1 and MycR-2 were hybridized to form the tandem Myc tag and were cloned *Bgl*II/*Avr*II into the pmorn1-YFP-MORN1/sagCAT plasmid [5]. Subsequently the plasmid was digested with *Not*I and the ends filled end in with T4 DNA polymerase followed by *Bgl*II digestion releasing the Myc_2_-MORN1-3′dhfr segment. This segment was cloned into pDHFR-Tet7sag1-*Spe*I by *Spe*I digestion, filling in the ends and subsequent *Bcl*I digestion to generate pTet7sag1Myc_2_-MORN1/DHFR (Eidell and Gubbels, manuscript in preparation).

The MORN1 open reading frame in cosmid PSBMG48 was replaced with a MORN1-flanking sequence flanked PCR amplified CAT cassette as previously described using primers MORN1_recomb-F and MORN1_recomb-R [23]. The recombineered cosmid was transfected into the conditional Tet7sag1-Myc2-MORN1 clone. Stable transfectants were cloned and screened for recombination of the MORN1 locus using primers MORN1_scr-F and MORN1_scr-R.

All MORN1 deletion plasmids were cloned based on the ptub-YFP_2_(MCS)/sagCAT plasmid [4]. Plasmids were generated by replacing a modular segment, either the α-tubulin promoter (*Pme*I/*Bgl*II), the first YFP (*Bgl*II/*Avr*II), the second YFP (*Avr*II/*Eco*RV), or the entire tandem YFP cassette (*Bgl*II/*Eco*RV). The sagCATsag cassette was replaced by the sagBLEsag cassette [48] by *Xho*I digest swapping. The megaprimer method was used to generate internal deletion or point mutations [49]. The basal IMCs plasmids were designed as follows: ptub-DD-YFP-IMCx/BLE, where x is either 5, 8 or 9 [4].

Plasmids used that have been described previously are ptub-H2b-mRFP/sagCAT [5], ptub-P30-YFP-HDEL/sagCAT and ptub-GRASP55-RFP/sagCAT (both a kind gift from Dr. Cynthia He, National U. of Singapore). Mitotracker-red staining was performed as described by [50].

### Fluorescence microscopy

Immunofluorescence assays (IFA) were performed as described previously [5]. Wild type parasites or parasites expressing tagged transgenes were grown in 6-well plates containing coverslips confluent with HFF cells and fixed with 100% methanol or 4% paraformaldehyde. The following specific primary antibodies were used: rabbit anti-IMC3 [51], rat anti-IMC3 [4], rabbit anti-MORN1 [5], MAb 45∶36 IMC1 (kindly provided by Dr. Gary Ward, Univ. Vermont), rabbit anti-human Centrin (kindly provided by Dr. Iain Cheeseman, Whitehead Institute), rabbit anti-GAP45 [kindly provided by Dr. Con Beckers, University of North Carolina; [52]], MAb 12G10 recognizing unmodified α-tubulin [Hybridoma Bank, University of Iowa; [53]]; MAb 5F5C5 α-Rop (kindly shared by Dr. Peter Bradley); MAb 6D10 α-Mic2 [kindly shared by Dr. David Sibley [54]]; MAb 9E10 α-cMyc (Invitrogen); rabbit α-GFP (Torrey Pines Biolabs); mouse α-GFP (Abgent); rabbit α-FKBP12 (Affinity BioReagents) recognizing the DD-domain [55]. Secondary α-rat, α-rabbit or α-mouse antibodies conjugated to Alexa488 or Alexa594 were used (Invitrogen). Nuclear material was co-stained with 4′,6-diamidino-2-phenylindole (DAPI). Images were collected on a Zeiss Axiovert 200 M wide-field fluorescence microscope equipped with standard DAPI, FITC, YFP, and TRITC filter sets, an α-Plan-Fluar 100×/1.45 NA oil objective and a Hamamatsu C4742-95 CCD camera. Images were collected, deconvoluted and adjusted for phase-contrast using Volocity software (Improvision).

Live cell time-lapse movies were acquired using an I×71 inverted epifluorescence microscope (Olympus) with a 100X oil immersion lens (UPlanApo 1.35 NA). Images were recorded using a Photometrics Coolsnap HQ camera and brightness/contrast were processed using SoftWoRx software (Applied Precision). Time-lapse imaging was performed in a humidified chamber heated to 37°C with 5% CO_2_, with cells grown in MatTek glass bottom culture dishes. Images were processed to account for cell drifting.

### Electron microscopy

For transmission electron microscopy (TEM), parasites were grown in HFF confluent T25 tissue culture flasks, released by trypsinization, and fixed with 2.5% glutaraldehyde in 0.1 M phosphate buffer pH 7.2. The samples were post-fixed in OsO_4_, dehydrated in ethanol, treated with propylene oxide and embedded in Spurr's epoxy resin. Sections were stained with uranyl acetate and lead citrate prior to examination in a JEOL 1200EX electron microscope [56].

For scanning electron microscopy (SEM), extracellular parasites were harvested following 48 hours of growth in the presence of ATc by filtration (polycarbonate, 12 µm pore size), washed twice in PBS and then fixed in 2.5% glutaraldehyde in 0.1 M phosphate buffer pH 7.4 at 4°C overnight. Fixed cells were settled onto poly-L-lysine coated coverslips, washed twice in 0.1 M phosphate buffer, post-fixed with 1% OsO_4_ and dehydrated through an ethanol series. The coverslips were critical-point dried and coated with 20 nm gold. Samples were examined with a JEOL JSM-6340F field emission scanning electron microscope.

### Western blotting

As described previously [5]. Primary antibodies as described above. Secondary antibodies were conjugated with horse radish peroxidase (DakoCytomation) and the signal visualized by ECL.

### DNA content analysis

Essentially as described previously [57]. Parasites were filtered through a 12 µm pore polycarbonate filter (Millipore) to allow detection of large, mutant parasites. DNA was stained with Sytox green and samples were analyzed on FACSCanto flow cytometer (Becton Dickinson) with FITC filter set. Data were analyzed and prepared for presentation using FloJo software (Treestar).

### Phenotype reversion assay

A T25 flask confluent with HFF cells was inoculated with 50–200 freshly lysed MORN1-KO parasites in Ed1 medium and incubated for 0, 12, 18, 24, 48, 72, or 168 (1 week) hrs with 1 µg/ml ATc in a humidified 5% CO_2_ incubator at 37°C. After these incubation times, cells were washed with 1× PBS, and Ed1 medium without ATc was added. 7–10 days later, the monolayer was stained with crystal violet to visualize plaques [47].

## Supporting Information

Table S1 Oligonucleotides used in this study. Restriction enzyme sites are underlined, point mutations are represented in underlined, bold font.
(0.03 MB DOC)

Figure S1 MORN1 knock-out strategy and identification of mutant clones. (A) MORN1 spanning cosmid PSBMG49 was recombineered to replace the MORN1 open reading frame with a gentamicin (for selection in E. coli) and CAT cassette (for selection in T. gondii). (B) The MORN1 replacement cosmid was transfected into a parasite clone expressing the Tet-transactivator and a Tet7sag1 promoter controlled Myc2-MORN1 construct (Fig. 1A–E). Twenty-one clones resistant to chloramphenicol were picked and checked for MORN1 replacement with the GENT/CAT cassette by PCR using the primers indicated in panel A. Highlighted clone C9 was used throughout this study.
(0.53 MB TIF)

Figure S2 Viability of the MORN1-KO phenotype upon ATc withdrawal after various times of ATc induction. (A) MORN1-KO parasites were allowed to plaque by inducing the phenotype for the indicated duration with ATc, followed by 9 days of growth in absence of ATc. Plaques were counted and plotted relative to the uninduced MORN1-KO strain. Average of three replicate experiments is shown; error bars denote standard deviation. Only 1 data point was collected at 96 hrs induction. (B) Stained plaque assays of the time course of ATc induction followed by 7 days plaque growth (0 hrs) or 9 days plaque growth (all others).
(2.39 MB TIF)

Figure S3 Single channel fluorescence panels corresponding with Figure 5 and additional IFAs to further illustrate that the basal IMC protein do not assemble into the basal complex in the MORN1-KO. (A–L) see legend Figure 5. (M,N) DD-YFP-IMC5 (Y-IMC5) and (O,P) DD-YFP-IMC9 (Y-IMC9) co-stained with DAPI (blue), and IMC3 antibodies (red). DD-tags were stained with α-FKBP12 (green). Arrowheads indicate the basal complex of some mature mothers (O) or the accumulation of DD-YFP-IMC5 or DD-YFP-IMC9 where the basal complex should have been assembled (N,P). Arrows in panel (M) indicate the contracting basal complex in two of the forming daughters, which are 3-fold enlarged in the inserts to visualize their circular appearance whereas in panel (M) the arrow points to correctly assembled DD-YFP-IMC9 in the basal complex. 1 µM Shield1 was added to stabilize the DD domain fusion proteins.
(10.05 MB TIF)

Figure S4 Dissection of the MORN1 overexpression phenotype by MORN1 deletion mutants. (A) Schematic representation of MORN1 and the tested deletion mutants. The MORN domains are numbered and their location indicated by green rectangles. The partial MORN motif at the N-terminus is labeled “PM” and the 5 amino acid linker region between MORN domains 6 and 7 is indicated with an arrowhead. The names of the constructs that were re-cloned under their endogenous promoter and without a tag for functional complementation studies are highlighted with a black background. (B) Summary of the observed YFP locations as outlined in panel C for the tested deletion constructs. Viability was determined by selecting for stable transfectants (three independent selection experiments were performed on all deletion constructs). (C) Representative images of the phenotypes that could be discerned. In several panels the parasites are co-transfected with H2b-RFP to identify the nucleus. WT: wild type; AG: aggregation (inclusion bodies); Fib: Fibers; CC: centrocone; AC: apical complex; iBC: immature basal complex; mBC: mature basal complex; Cyt: cytoplasm. Arrows and arrowheads as indicated in the panel. All MORN1 constructs were driven by the α-tubulin promoter and at the C-terminus fused to YFP. All microscopy was performed on live parasites.
(1.71 MB TIF)

Figure S5 Single channels fluorescence images of Figure 7D–K. For legend see Figure 7.
(6.52 MB TIF)

Movie S1 MORN1-KO parasites expressing YFP-IMC3 and FNR-RFP were induced for 12 hrs with ATc and then imaged for 14 hrs. Time points are indicated where every time point represents a 10 min lapse.
(2.12 MB MOV)
