# Supplementary material for: A Toxoplasma MORN1 Null Mutant Undergoes Repeated Divisions but Is Defective in Basal Assembly, Apicoplast Division and Cytokinesis
Source: PLoS One. 2010 Aug 19;5(8):e12302. doi: 10.1371/journal.pone.0012302 (PMC2924399; doi:10.1371/journal.pone.0012302)
Supplement: Table S1 — Oligonucleotides used in this study. Restriction enzyme sites are underlined, point mutations are represented in underlined, bold font. (0.03 MB DOC) [file pone.0012302.s001.doc]

**Supplementary Table S1. Oligonucleotides used in this study.** Restriction enzyme sites are underlined, point mutations are represented in underlined, bold font.

_____________________________________________________________________________

Primer name Sequence

_____________________________________________________________________________

Myc F-1 GATCTAAAATGGAACAAAAGCTAATCTCCGAGGAAGACTTGAACG

Myc F-2 GTGCTAGGGCCGAGGAGCAGAAGCTGATCTCCGAGGAGGACCTGC

Myc R-1 CCTAGCACCGTTCAAGTCTTCCTCGGAGATTAGCTTTTGTTCCATTTTA

Myc R-2 CTAGGCAGGTCCTCCTCGGAGATCAGCTTCTGCTCCTCGGC

BglII-F-MORN1.1 CAGAGATCTAAAATGGAGAGCTGCCACGCG

BglII-F-MORN1.2 CAGAGATCTAAAATGTACGAGGGGGAGTTCGTGTTC

BglII-F-MORN1.3 CAGAGATCTAAAATGTACGAAGGGAAGTGGGTCGAAG

BglII-F-MORN1.5 CAGAGATCTAAAATGTACGAGGGCGAGTGGGTGG

BglII-F-MORN1.7 CAGAGATCTAAAATGTACGAGGGCGACTGGGTGAAC

BglII-F-MORN1.9 CAGAGATCTAAAATGTACGAAGGCGAGTGGGCTC

BglII-F-MORN1.11 CAGAGATCTAAAATGTACGAAGGCGAATGGACAG

BglII-F-MORN1.12 CAGAGATCTAAAATGTTCAAAGGCCAGTGGGCCGAC

BglII-F-MORN1.13 CAGAGATCTAAAATGTACGAAGGCGAATGGACAG

BglII-F-MORN1.PM CAGAGATCTAAAATGCTCGAAGGCACCTGGTC

AvrII-F-MORN1.1 CAGCCTAGGATGGAGAGCTGCCACGCG

AvrII-F-MORN1.5 CAGCCTAGGATGTACGAGGGCGAGTGGGTGG

AvrII-F-MORN1.7 CAGCCTAGGATGTACGAGGGCGACTGGGTGAAC

AvrII-R-MORN1.3 CAGCCTAGGCCGGTTGCCACTGGCGAAATG

AvrII-R-MORN1.5 CAGCCTAGGGACATCGCCCTCCGCGTAG

AvrII-R-MORN1.6 CAGCCTAGGACCTTTGGCACTGACGTACGTC

AvrII-R-MORN1.8 CAGCCTAGGCACGTTGCCGTTCGGAAACAC

AvrII-R-MORN1.10 CAGCCTAGGCTTGTCACCGCGGGTGTAAG

AvrII-R-MORN1.12 CAGCCTAGGTCGGTTCCCGTTTGCATACGTG

AvrII-R-MORN1.14 CAGCCTAGGTTGGTGGCCAGTCGCGAG

AvrII-R-MORN1.PM CAGCCTAGGCAAGTCGACATTGAGCCATG

RV-R-MORN1.6 CAGGATATCTTAACCTTTGGCACTGACGTAC

RV-R-MORN1.8 CAGGATATCTTACACGTTGCCGTTCGGAAACAC

RV-R-MORN1.10 CAGGATATCTTACTTGTCACCGCGGGTGTAAG

RV-R-MORN1.12 CAGGATATCTTATCGGTTCCCGTTTGCATACGTG

RV-R-MORN1.PM CAGGATATCTTACAAGTCGACATTGAGCCATG

C4A MORN1-Bgl-F CAGAGATCTAAAATGGAGAGC**GC**CCACGCGTACCACGGACAG

MORN1-DelLink-R GTTCACCCAGTCGCCCTCGTAACCTTTGRCACTGACGTACGT

MORN1_recomb-F ATGCAAGCCGCGTACACTTCATTCCCCGTTTTTCCTTACCGTTGTCCACCATA

CGACTCACTATAGGGCGAATTGG

MORN1_recomb-R  TGAAACAGACCGTCTTTGATCTGTCCGTGGTACGCGTGGCAGCTCTCCATTTT

GGTTGAAGACAGACGAAAGCAGTTG

MORN1_scr-F  TTCTCACTGTATCTCCTGTCTTG

MORN1_scr-R  TACTCTGCCGCATGGATAAGC

_______________________________________________________________________
