# Supplementary figures and images for: A Toxoplasma MORN1 Null Mutant Undergoes Repeated Divisions but Is Defective in Basal Assembly, Apicoplast Division and Cytokinesis
Source: PLoS One. 2010 Aug 19;5(8):e12302. doi: 10.1371/journal.pone.0012302 (PMC2924399; doi:10.1371/journal.pone.0012302)

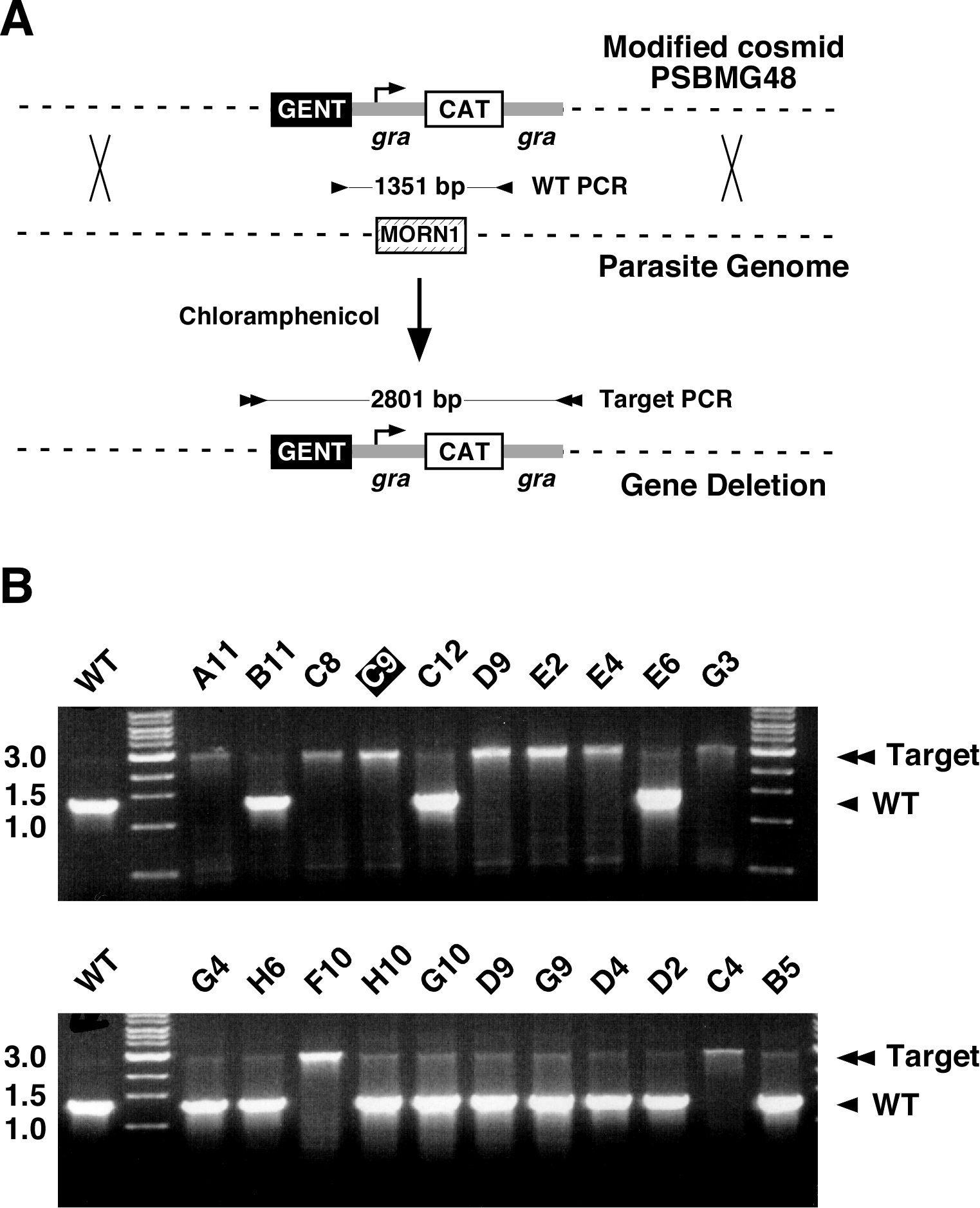

Supplement: Figure S1 — MORN1 knock-out strategy and identification of mutant clones. (A) MORN1 spanning cosmid PSBMG49 was recombineered to replace the MORN1 open reading frame with a gentamicin (for selection in E. coli) and CAT cassette (for selection in T. gondii). (B) The MORN1 replacement cosmid was transfected into a parasite clone expressing the Tet-transactivator and a Tet7sag1 promoter controlled Myc2-MORN1 construct (Fig. 1A–E). Twenty-one clones resistant to chloramphenicol were picked and checked for MORN1 replacement with the GENT/CAT cassette by PCR using the primers indicated in panel A. Highlighted clone C9 was used throughout this study. (0.53 MB TIF) [file pone.0012302.s002.tif]

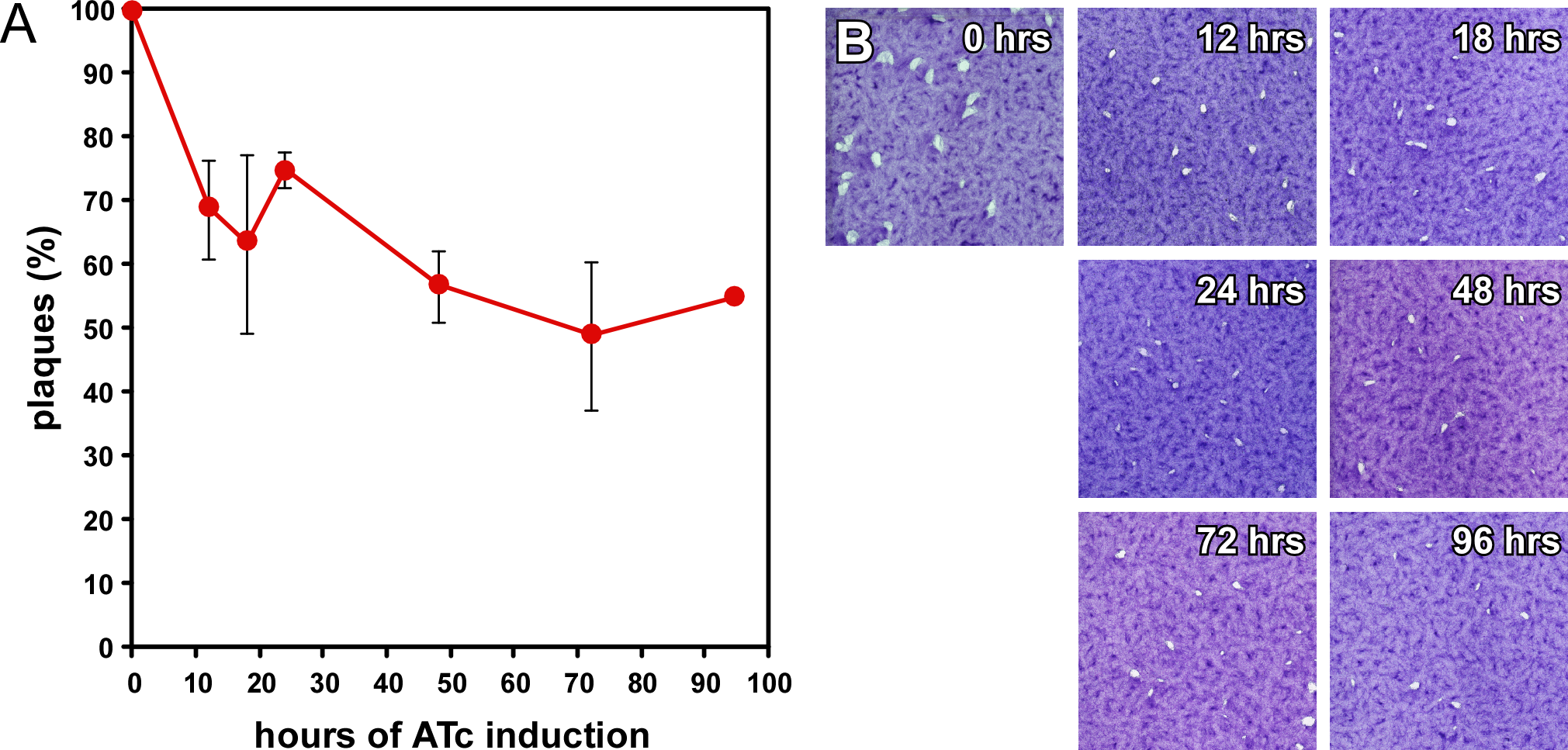

Supplement: Figure S2 — Viability of the MORN1-KO phenotype upon ATc withdrawal after various times of ATc induction. (A) MORN1-KO parasites were allowed to plaque by inducing the phenotype for the indicated duration with ATc, followed by 9 days of growth in absence of ATc. Plaques were counted and plotted relative to the uninduced MORN1-KO strain. Average of three replicate experiments is shown; error bars denote standard deviation. Only 1 data point was collected at 96 hrs induction. (B) Stained plaque assays of the time course of ATc induction followed by 7 days plaque growth (0 hrs) or 9 days plaque growth (all others). (2.39 MB TIF) [file pone.0012302.s003.tif]

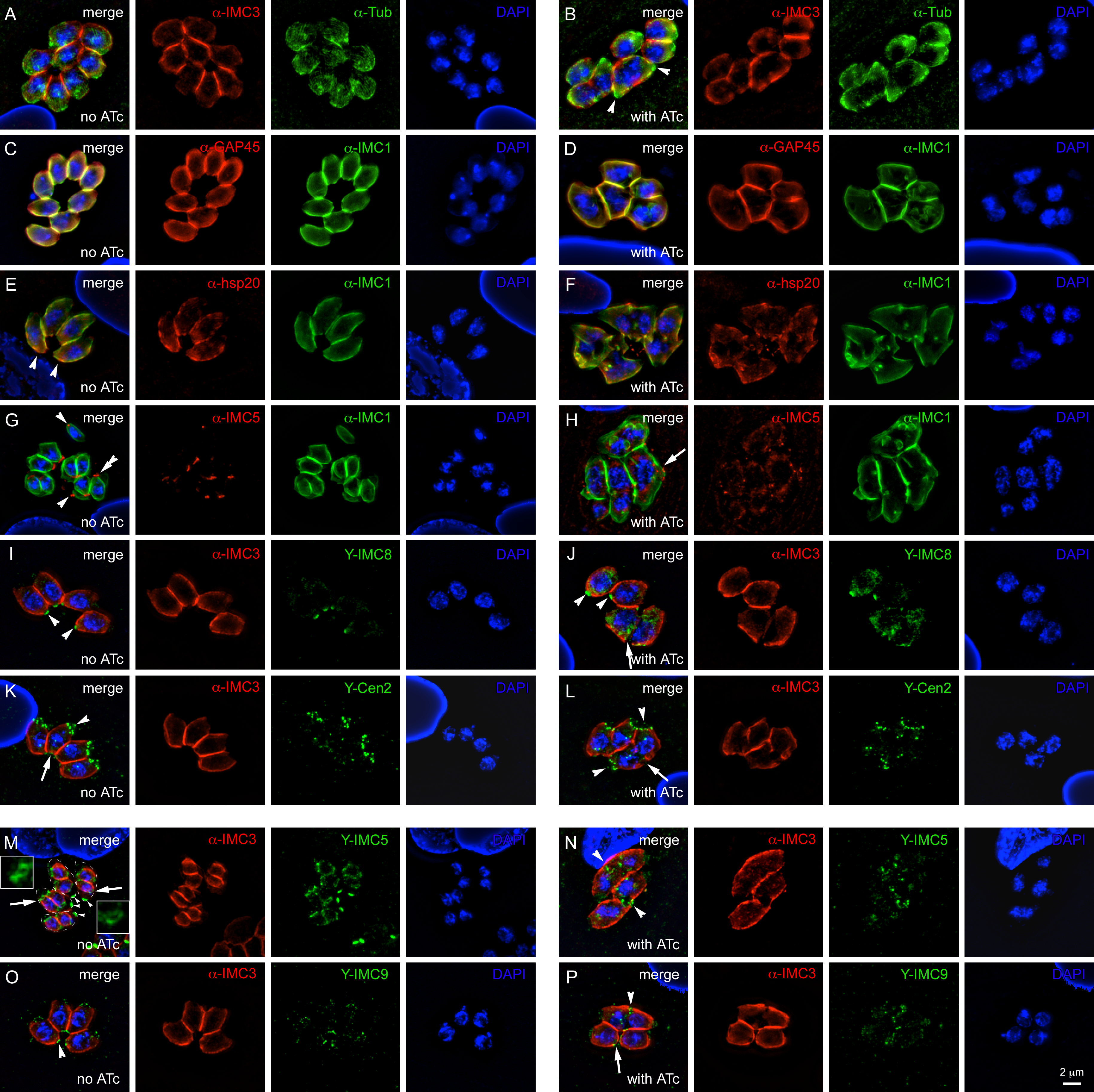

Supplement: Figure S3 — Single channel fluorescence panels corresponding with Figure 5 and additional IFAs to further illustrate that the basal IMC protein do not assemble into the basal complex in the MORN1-KO. (A–L) see legend Figure 5. (M,N) DD-YFP-IMC5 (Y-IMC5) and (O,P) DD-YFP-IMC9 (Y-IMC9) co-stained with DAPI (blue), and IMC3 antibodies (red). DD-tags were stained with α-FKBP12 (green). Arrowheads indicate the basal complex of some mature mothers (O) or the accumulation of DD-YFP-IMC5 or DD-YFP-IMC9 where the basal complex should have been assembled (N,P). Arrows in panel (M) indicate the contracting basal complex in two of the forming daughters, which are 3-fold enlarged in the inserts to visualize their circular appearance whereas in panel (M) the arrow points to correctly assembled DD-YFP-IMC9 in the basal complex. 1 µM Shield1 was added to stabilize the DD domain fusion proteins. (10.05 MB TIF) [file pone.0012302.s004.tif]

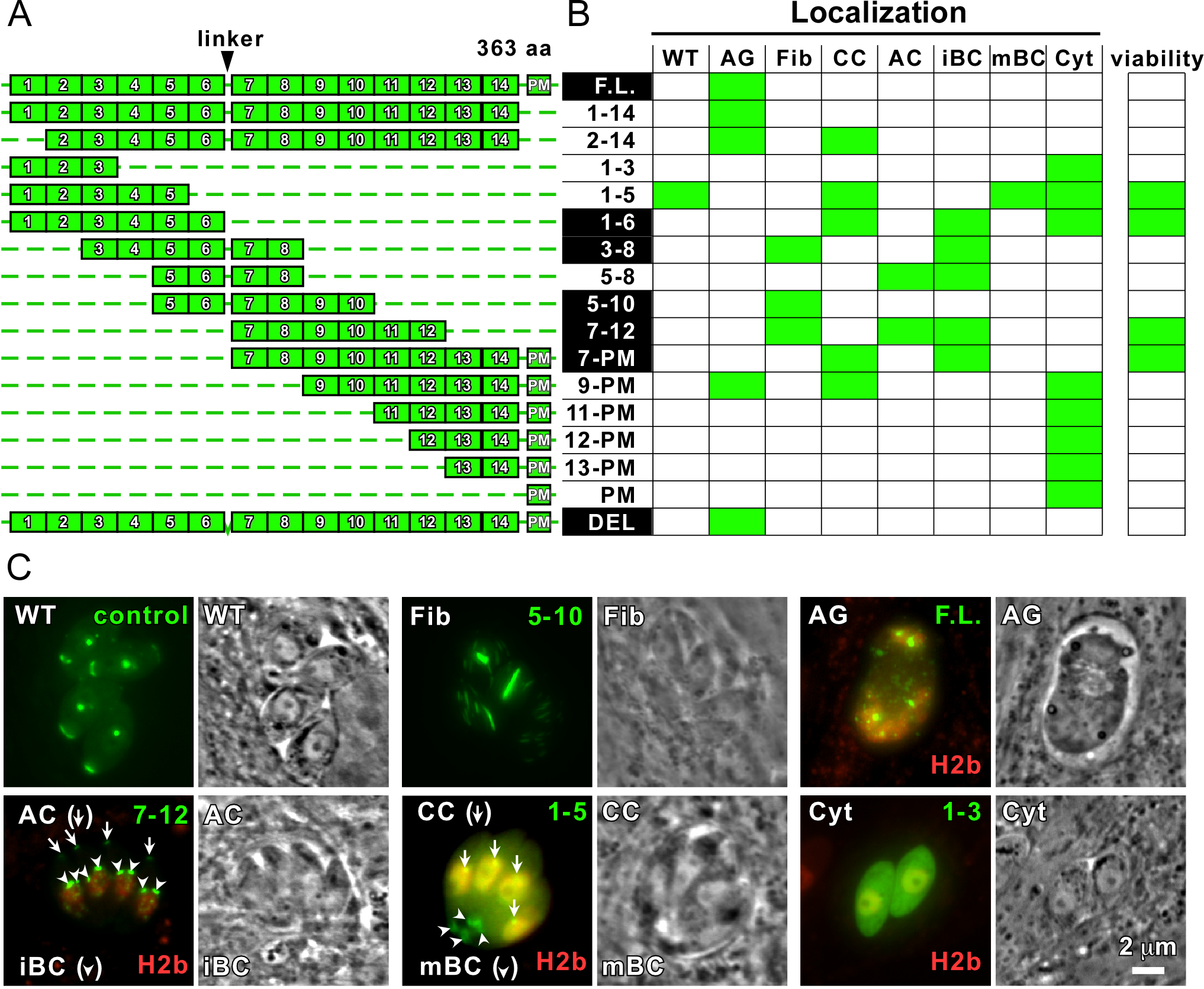

Supplement: Figure S4 — Dissection of the MORN1 overexpression phenotype by MORN1 deletion mutants. (A) Schematic representation of MORN1 and the tested deletion mutants. The MORN domains are numbered and their location indicated by green rectangles. The partial MORN motif at the N-terminus is labeled “PM” and the 5 amino acid linker region between MORN domains 6 and 7 is indicated with an arrowhead. The names of the constructs that were re-cloned under their endogenous promoter and without a tag for functional complementation studies are highlighted with a black background. (B) Summary of the observed YFP locations as outlined in panel C for the tested deletion constructs. Viability was determined by selecting for stable transfectants (three independent selection experiments were performed on all deletion constructs). (C) Representative images of the phenotypes that could be discerned. In several panels the parasites are co-transfected with H2b-RFP to identify the nucleus. WT: wild type; AG: aggregation (inclusion bodies); Fib: Fibers; CC: centrocone; AC: apical complex; iBC: immature basal complex; mBC: mature basal complex; Cyt: cytoplasm. Arrows and arrowheads as indicated in the panel. All MORN1 constructs were driven by the α-tubulin promoter and at the C-terminus fused to YFP. All microscopy was performed on live parasites. (1.71 MB TIF) [file pone.0012302.s005.tif]

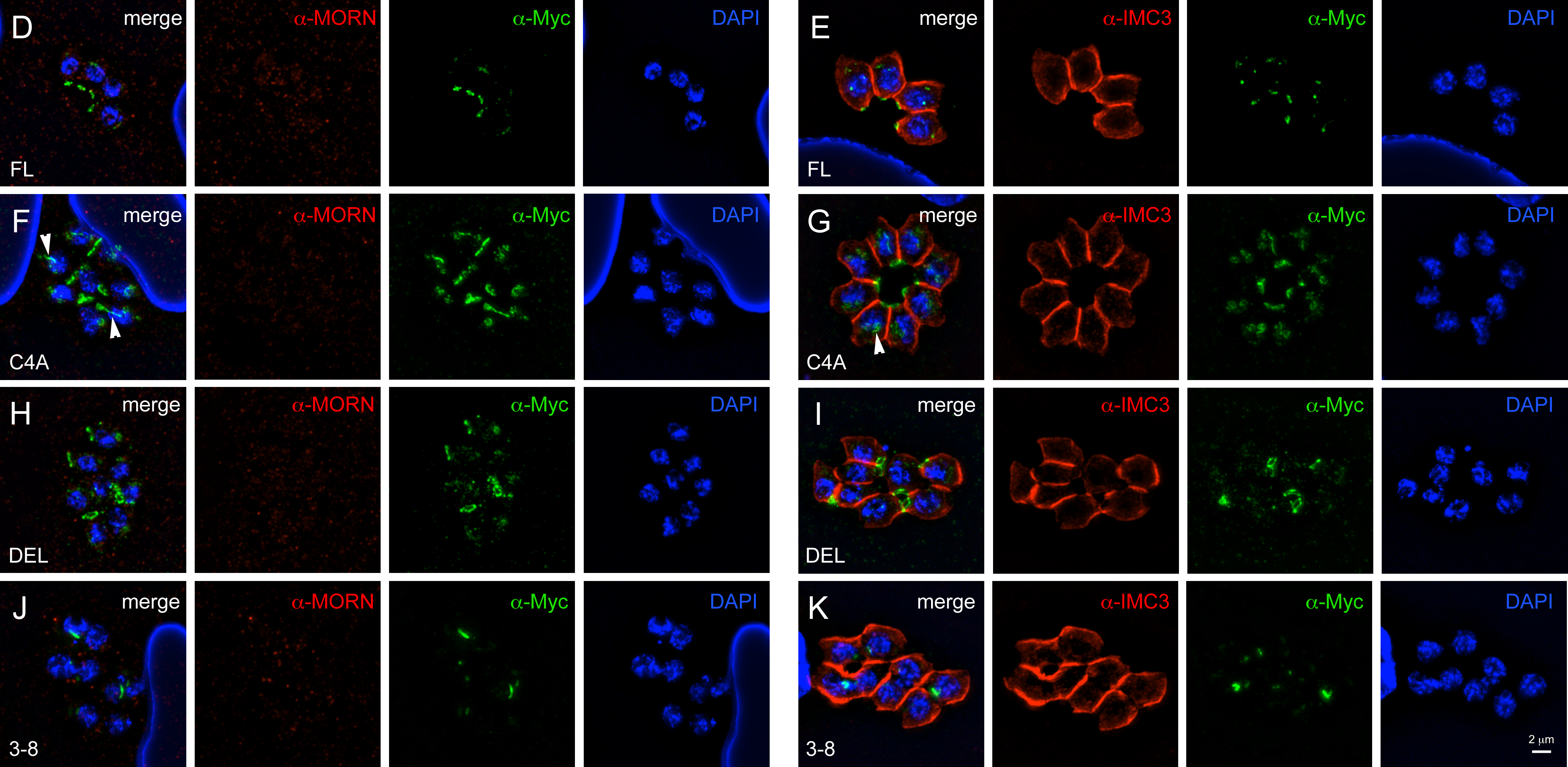

Supplement: Figure S5 — Single channels fluorescence images of Figure 7D–K. For legend see Figure 7. (6.52 MB TIF) [file pone.0012302.s006.tif]
